# Integrating Mobile and Fixed-Site Black Carbon Measurements to Bridge Spatiotemporal Gaps in Urban Air Quality

**DOI:** 10.1021/acs.est.3c10829

**Published:** 2024-07-01

**Authors:** Chirag Manchanda, Robert A. Harley, Julian D. Marshall, Alexander J. Turner, Joshua S. Apte

**Affiliations:** † Department of Civil and Environmental Engineering, University of California, Berkeley, California 94720, United States; ‡ Department of Civil and Environmental Engineering, University of Washington, Seattle, Washington 98195, United States; § Department of Atmospheric Sciences, University of Washington, Seattle, Washington 98195, United States; ∥ School of Public Health, University of California, Berkeley, California 94720, United States

**Keywords:** black carbon, spatiotemporal modeling, mobile monitoring, low-cost sensors, hyperlocal, urban air quality

## Abstract

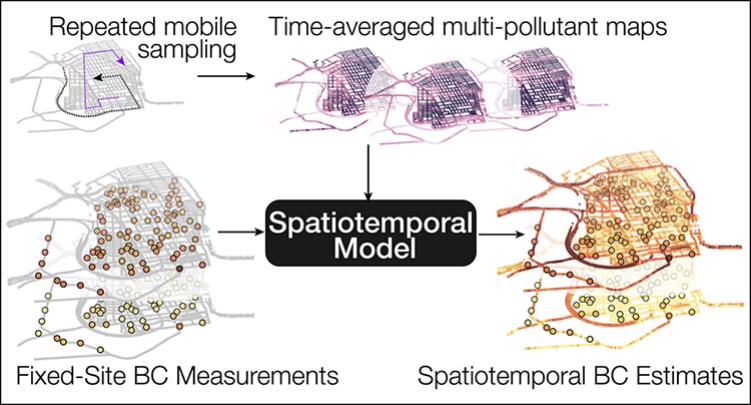

Urban air pollution can vary sharply in space and time. However, few monitoring strategies can concurrently resolve spatial and temporal variation at fine scales. Here, we present a new measurement-driven spatiotemporal modeling approach that transcends the individual limitations of two complementary sampling paradigms: mobile monitoring and fixed-site sensor networks. We develop, validate, and apply this model to predict black carbon (BC) using data from an intensive, 100-day field study in West Oakland, CA. Our spatiotemporal model exploits coherent spatial patterns derived from a multipollutant mobile monitoring campaign to fill spatial gaps in time-complete BC data from a low-cost sensor network. Our model performs well in reconstructing patterns at fine spatial and temporal resolution (30 m, 15 min), demonstrating strong out-of-sample correlations for both mobile (Pearson’s *R* ∼ 0.77) and fixed-site measurements (*R* ∼ 0.95) while revealing features that are not effectively captured by a single monitoring approach in isolation. The model reveals sharp concentration gradients near major emission sources while capturing their temporal variability, offering valuable insights into pollution sources and dynamics.

## Abstract 2

We develop, test, and demonstrate a new model that combines stationary and mobile air pollution measurements to predict how air pollution varies in space and time at very high resolution.

## 1 Introduction

Air pollution adversely impacts public health and the environment.^1−3^ Owing to the interplay of atmospheric dynamics and unevenly distributed sources, air pollution can vary sharply in space and time.^4−8^ While conventional air pollution measurements are sparse in space and/or time, recognizing the historically high cost of acquiring measurements, spatiotemporally resolved air pollution data are increasingly in demand. This need arises from the desire to gain detailed insights into the sources and processes in urban environments, address societal impacts such as exposures and inequalities, and facilitate effective management strategies.^9−11^

There has been a surge of interest in hyperlocal air pollution monitoring, including mobile monitoring^7,8,12−27^ and fixed-site low-cost sensor (LCS) networks.^7,28^ These methods can overcome the limitations of traditional monitoring to better capture the fine-scale structure of air pollution in urban settings but have their own advantages and drawbacks.^7^ Mobile measurements can provide high spatial resolution to quantify fine-scale concentration gradients, thereby identifying previously undiscovered pollutant sources and hotspots. However, they have intermittent temporal coverage, resulting in data that may miss critical pollution events. In contrast, like their regulatory monitoring counterparts, fixed-site LCS networks typically provide temporally complete data. However, despite considerably higher spatial density in comparison to regulatory monitoring, LCS networks often still have large spatial gaps compared to the street-level resolution attainable through mobile monitoring. Here, we present and explore a new method for capitalizing on the complementary strengths of these two hyperlocal monitoring paradigms.

Previous studies have explored methods to address the limitations of these monitoring strategies.^29^ These approaches include land-use regression (LUR),^10,18,30−33^ kriging methods,^10,34^ and a combination of other statistical learning techniques.^4,13,21,30,34,^ However, few studies have explored combining fixed-site and mobile measurements into a single spatiotemporal data product. Adams and Kanaroglou^36^ utilized a neural network-LUR framework to combine stationary and mobile PM_2.5_ measurements with land-use covariates and meteorology to develop spatially dense hourly estimates of PM_2.5_. Following a similar approach, Simon et al.^37^ employed mobile measurements to calculate spatial enhancements in UFP concentration in relation to a stationary monitor, subsequently integrating these enhancements into a regression model. However, very few studies have attempted to integrate stationary and mobile pollutant measurements into a fully measurement-driven spatiotemporal model, that is, a model relying exclusively on observations as inputs without additional predictor variables.

Our objective here is to describe, validate, and apply a new spatiotemporal modeling methodology for capturing fine-scale spatiotemporal variation in black carbon (BC) by fusing data from a LCS network and mobile monitoring. By leveraging information from coherent multipollutant spatial patterns measured by mobile monitoring, we effectively reveal nuanced spatiotemporal patterns in BC that are not readily apparent in either underlying data set.

## 2 Materials and Methods

### 2.1 Measured Data and Study Area

Our modeling approach entails fusing *time-resolved* but spatially sparse fixed-site data from low-cost sensors with *time-averaged* but spatially dense maps from mobile monitoring. The approach allows us to develop a BC model at high spatial (30 m) and temporal (15 min) resolution. To do so, we exploit an unusually rich data set of mobile- and fixed-site measurements collected in West Oakland (WO), California.

During a 100 day period from 19th May to 27th August 2017, Caubel et al.^28^ deployed a BC measurement network consisting of 100 sensors (“100 × 100 BC network”) distributed over an area of 15 km^2^ in WO. This network relied on a custom low-cost sensor (LCS), the Aerosol Black Carbon Detector (ABCD), which functions similarly to an aethalometer.^38,39^ The 100 LCS sites were distributed across residential, industrial, and high-traffic microenvironments. These data were recently used by Wai et al.^40^ in a separate effort to develop a spatiotemporal BC model. We focus here on 97 sites located within 30 m of the road network covered by mobile monitoring. The LCS sensors natively report BC at a resolution of 0.5 Hz.^28^ We systematically experimented with multiple LCS averaging times between 1 and 120 min, ultimately selecting a time resolution of 15 min to balance between preserving spatial heterogeneity and reducing instrument noise (see Supporting Information (SI) Section S1.1 and Figure S1 for details).^7^

In addition, the LCS campaign coincided with an extensive ongoing mobile monitoring effort that used two custom-equipped Google Street View cars, which sampled repeatedly on every city block of WO during 2015–2018. As described by Chambliss et al.^41^ and references therein, the vehicles recorded instantaneous (1 Hz) measurements of GPS location and concentrations of BC and other pollutants (NO, NO_2_, ultrafine particles [UFP], and 6 size-resolved particle concentrations bins from 0.3 to 10 μm).^7,12,18^ BC was measured using photoacoustic extinctiometers (PAX, Droplet Measurement Technologies, Longmont, CO);^42^ see SI Section S1.2 for a description of the full measurement suite for other species. Chambliss et al.^41^ found strong instrument–instrument agreement between the two mobile PAX instruments and between the PAX and the fixed-site ABCD sensors (PAX–PAX comparison: Pearson *R*^2^ = 0.97 and NRMSE = 0.15; PAX–ABCD comparison: Pearson *R*^2^ = 0.90 and NRMSE = 0.33).

For our core analysis, we use mobile monitoring data collected between 6 AM and 8 PM on 49 days within the 100 day period that the LCS sensor network operated. This data set emphasizes weekday, daytime conditions (70% on weekdays; 71% between 9 am and 4 pm). In previous work, Apte et al.^12^ reported that 10–20 repeat drive passes were sufficient to reproduce key spatial patterns with good precision and minimal bias. Here, we restrict the spatial domain to those roads with a minimum of 15 repeated drive visits (median visits = 31). The ∼150k 1-Hz time-resolved measurements were aggregated to “median-of-drive-pass-mean” concentrations for ∼4300 30-m-long road segments following the approach of Messier et al.^18^ That step resulted in 10 time-integrated maps for BC and the 9 other measured pollutants. This suite of multipollutant measurements played an essential role in developing the spatiotemporal model for BC.

### 2.2 Spatiotemporal Modeling Framework

#### 2.2.1 Conceptual Framework

The multifaceted interplay governing air pollution unfolds across three fundamental dimensions: location, time, and pollutant composition. However, most air quality monitoring strategies cannot capture the three dimensions simultaneously. Rather, dominant monitoring paradigms tend to emphasize at most two dimensions. For example, regulatory monitoring systems generally provide continuous measurements of multiple pollutants but only at a small number of distinct locations, thus constituting a *pollutant*-*time* system. Maps derived from repeated mobile measurements frequently yield time-integrated data for many pollutants at high spatial density, thus representing a *pollutant*-*location* system. LCS networks excel at providing spatial coverage at high time resolution, constituting a *location*-*time* system, but generally have a more limited capacity to measure detailed chemical speciation. Similar to a few other recent data-driven air pollution studies,^43,44^ our modeling framework is inspired by a common signal processing technique called compressive sensing (SI Section S1.3).^45−47^ Unlike more conventional spatiotemporal air pollution modeling methods, this approach relies exclusively on observations as inputs and does not require spatial or temporal predictor variables (e.g., land-use data, meteorology).^30−32,48^

Matrix factorization techniques (e.g., principal component analysis [PCA], non-negative matrix factorization [NMF]) are widely used in air quality research.^49−52^ These techniques exploit coherence across spatial, temporal, or chemical variability to uncover common pollution sources or meteorological conditions concurrently impacting these dimensions. Matrix factorizations are routinely applied to *pollutant*-*time* systems to leverage covariance among multiple pollutant time series to apportion each pollutant to one or more underlying contributing factors or sources represented by a set of covarying pollutants.^49^ The same techniques can also be extended to *pollutant*-*location*^53^ and *location*-*time*^50^ systems.

Our approach is grounded in the assumption that if common sources or meteorological conditions influence the spatial distribution of multiple pollutants across several time scales, information captured along one dimension can be leveraged to bridge gaps along another dimension. Here, we use the spatial variability captured by time-averaged multipollutant mobile measurements to fill spatial gaps between fixed-site BC measurements. At its core, the model decomposes a *pollutant-location* system, represented by mobile measurement-derived maps for multiple pollutants, and a *location-time* system, represented by continuous fixed-site BC measurements. This decomposition allows for the extraction of spatial patterns that repeat across multiple pollutants and across different time scales, respectively. By decomposing the input multipollutant maps (*pollutant-location)* into a smaller set of repetitive patterns, we are able to express each map as a weighted combination of these patterns (see SI Section S1.4 and Figure S2). Likewise, we decompose the 97 unique LCS time series (*pollutant-time*) into a smaller set of temporal patterns, with each site’s time series expressed as a weighted combination of these patterns. Given that the same meteorological conditions and emission sources influence both sets of patterns, the model establishes a best-fit relationship between them. Consequently, the model utilizes the more comprehensive multipollutant spatial patterns from mobile measurements to fill in the spatial gaps in BC observed in the sparser patterns from fixed-site measurements.

#### 2.2.2 Mathematical Model

**Figure 1:**
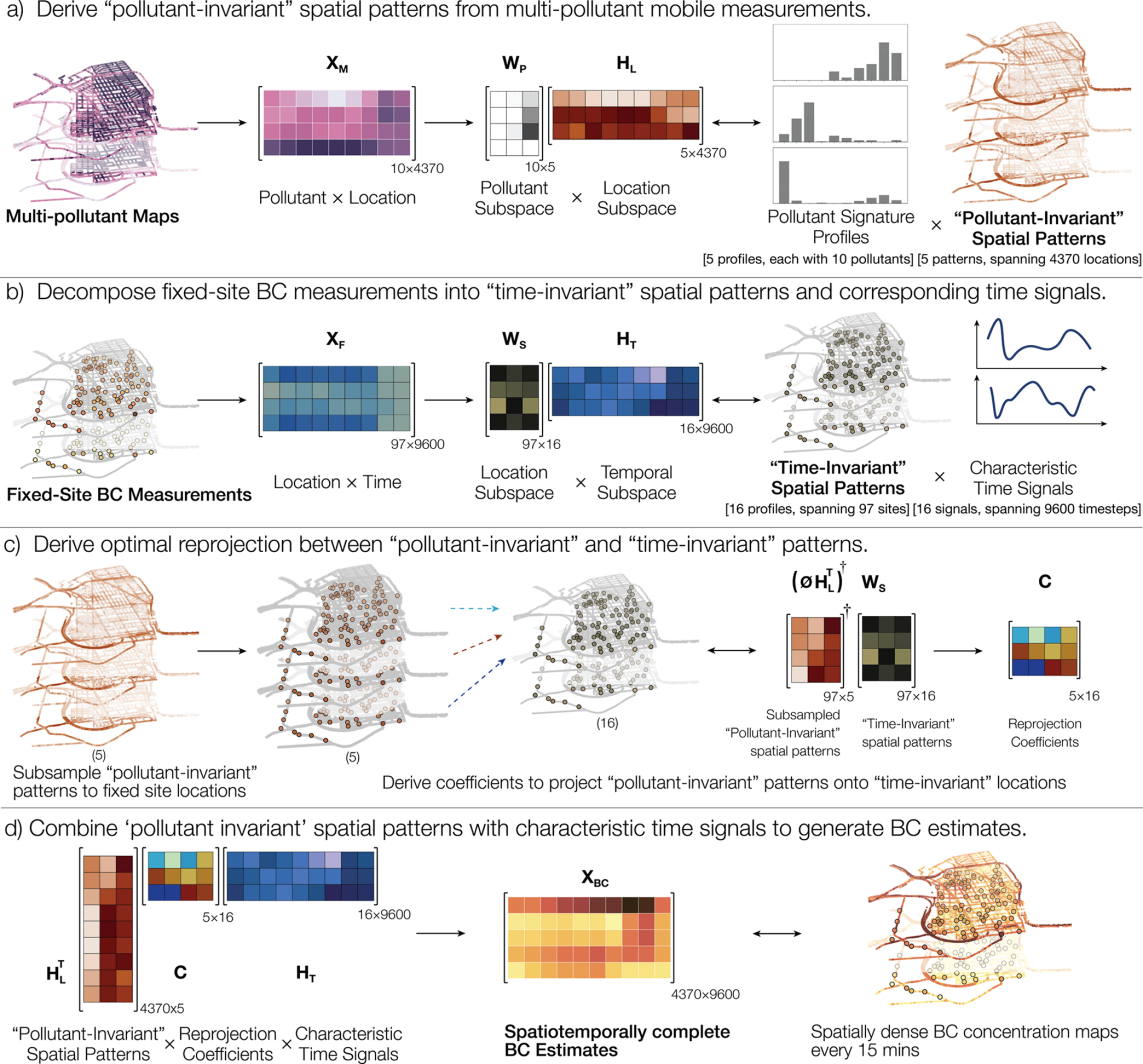
Schematic of a spatiotemporally complete model of BC based on the fusion of mobile and fixed-site data sets collected contemporaneously over 100 days in the Summer of 2017. Conceptually, the approach uses multipollutant information from (a) mobile monitoring, which produces time-averaged but spatially complete multipollutant maps, to fill in spatial gaps in a (b) sparser network of 97 fixed-site BC monitors that provide temporally complete data. To do so, we use matrix factorization (a) to represent time-averaged mobile monitoring maps for 10 pollutants as a linear combination of 5 factors with each factor having its own richly detailed spatial map (“pollutant-invariant spatial patterns”). Likewise, we decompose (b) the 100-day temporal variation of 15 min data (9600 observations) at fixed sites into 16 characteristic time signals. The relative weighting of these signals over all sites forms 16 “time-invariant spatial patterns”. In part (c), we derive an optimal reprojection matrix that links the 5 spatially dense multipollutant spatial patterns from part (a) to the 16 spatially sparser temporal patterns from part (b). Finally, these reprojection coefficients are employed to develop a spatially dense temporally continuous model for BC **(d)** that provides 9600 time estimates (100 days with 15 min data) over a map of 4370 locations in our measurement domain.

Figure 1 provides a schematic illustration outlining our approach. Figure 1a highlights how the multipollutant maps can be represented as a *pollutant × location* matrix, say **X**_***M***_ ∈ *R*^p×l^, with each matrix element representing time-averaged concentrations of the *p*^*th*^ pollutant and *l*^*th*^ location. In this study, *p* = 10 pollutants and *l* = 4370 locations (i.e., 4273 road segments plus 97 LCS network sites; we extended the pollutant measurements from the road segments to the nearby LCS sites using ordinary kriging).^53^ This matrix **X**_***M***_ can now be factorized into a pollutant subspace **W**_***P***_ and a location subspace **H**_***L***_, using NMF as follows
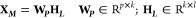
1Drawing on analogy to the concept of *pollutant*-*time* source apportionment, NMF applied to this *pollutant*-*location matrix* apportions the spatial variation in concentration to *k*-source profiles. In the present case, using the knee-point method, we found a five-factor solution (i.e., *k* = 5) to be optimal for describing the input data (See SI Section S1.5 and Figure S1 for details). **W**_***P***_ represents the fractional abundance of the *p*^*th*^ pollutant in the *k*^*th*^ source, and thus, each column vector in **W**_***P***_ represents a source profile or source signature (Figure 1a). **H**_***L***_ represents the normalized concentration attributable to the *k*^*th*^ source at the *l*^*th*^ location, and thus, each row in **H**_***L***_ represents a *pollutant*-*invariant* spatial pattern. Analogous to how *pollutant*-*time* source apportionment yields source profiles and corresponding time signals, each *pollutant*-*invariant* spatial pattern describes the impact corresponding to each derived source profile. While these source profiles may contain interpretable information, here, our focus is on using their spatial signatures to translate coherent patterns from one dimension to another.

The second component of the spatiotemporal model consists of the BC measurements obtained from the LCS network. Figure 1b represents the fixed-site BC measurements as a *location × time* matrix, say **X**_***F***_∈R^x×t^. For the current study, we have 97 LCS sites (*s*). We averaged measurements at 15 min time resolution over the 100 day study duration, resulting in 9600 timesteps (*t*). **X**_***F***_ can similarly be decomposed into a spatial (**W**_***S***_) and temporal (**H**_***T***_) subspace using NMF as follows:
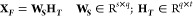
2Through systematic exploration, we determined that the most suitable value for *q* was 16 factors (See SI Section S1.5 and Figure S1 for details.) Here, each of the *q* = 16 rows of **H**_***T***_ represents one of the 16 characteristic time signals, and each of the columns **W**_***S***_ represents a spatial pattern that remains invariant for that corresponding signal, thus a *time*-*invariant* BC concentration field or *time-invariant spatial pattern* (Figure 1b). These patterns denote sets of spatial points that covary according to the same time signal. While we do not focus here on the interpretability of these patterns, they may reflect diurnal factors such as traffic or industries or meteorological conditions like wind patterns or urban infrastructure impacts, affecting similar locations in a similar manner. The NMF-based decomposition of the mobile and fixed-site data yields two matrices, **H**_***L***_ and **W**_***S***_, corresponding to a set of *pollutant*-*invariant* and *time*-*invariant* spatial patterns, respectively. Revisiting our initial assumption, if we posit that common pollution sources and meteorological conditions influence the spatial distribution of pollutant concentrations consistently over time, then these *pollutant*-*invariant* and *time*-*invariant* spatial patterns essentially portray different perspectives of the same underlying reality. Consequently, they can be reciprocally reprojected, effectively expressing one in terms of the other. In other words, each pattern in one of the sets can be expressed as a combination of patterns in the other set.

Figure 1c illustrates this reprojection process. It starts by taking a transpose of *pollutant*-*invariant* location subspace **H**_***L***_^***T***^ ∈ R^l×k^ and subsampling it to the locations where fixed-site measurements are available, using a binary sampling matrix ⌀ ∈ R^s×l^, resulting in ⌀**H**_***L***_^***T***^ ∈ R^s×k^, which represent the 5 *pollutant*-*invariant* patterns at the 97 LCS sites, i.e., the overlap between the *pollutant*-*invariant* and time-invariant patterns. This subsampled matrix subsequently undergoes a Moore*–*Penrose Inverse with respect to the *time*-*invariant* location subspace **W**_***S***_ ∈ R^s×q^, yielding a coefficient matrix **C** ∈ R^k×q^. Each column of this coefficient matrix signifies the best-fit coefficients for representing each of the 16 time-invariant patterns (derived from eq 2) as a combination of the 5 pollutant-invariant patterns such that
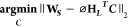
3The matrices ⌀**H**_***L***_^***T***^ ∈ R^97×5^ and **W**_***S***_ ∈ R^97×16^ represent the *pollutant-invariant* patterns and the *time-invariant* patterns at the set of spatial points where both patterns overlap: the LCS network sites (Figure 1c). The coefficient matrix ***C*** ∈ R^5×16^ effectively maps one set onto the other. While we derived ***C*** using an overlapping subset between the time-invariant and pollutant-invariant patterns, the *pollutant-invariant* set **H**_***L***_^***T***^ ∈ R^4370×5^ possesses denser spatial information. Leveraging ***C***, we can now “fill in the gaps” by combining the *pollutant-invariant* patterns using ***C*** to generate spatially augmented versions of the *time-invariant* patterns, expressed as **H**_***L***_^***T***^***C*** ∈ R^4370×16^. These augmented patterns are finally multiplied with the corresponding characteristic time signals, **H**_***T***_ ∈ R^16×9600^ (eq 4), as shown in Figure 1d, to generate the desired model output: spatiotemporally complete BC estimates.
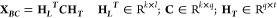
4The matrix **X**_***BC***_ represents the resultant model estimates of BC integrating the spatial density of mobile measurements and temporal completeness of the fixed-site LCS measurements. In essence, the model leverages dense spatial patterns observed across multiple pollutants to fill gaps in sparser spatial patterns repeating over time to estimate a spatiotemporally complete BC surface.

### 2.3 Core Model and Sensitivity Cases

**Figure 2:**
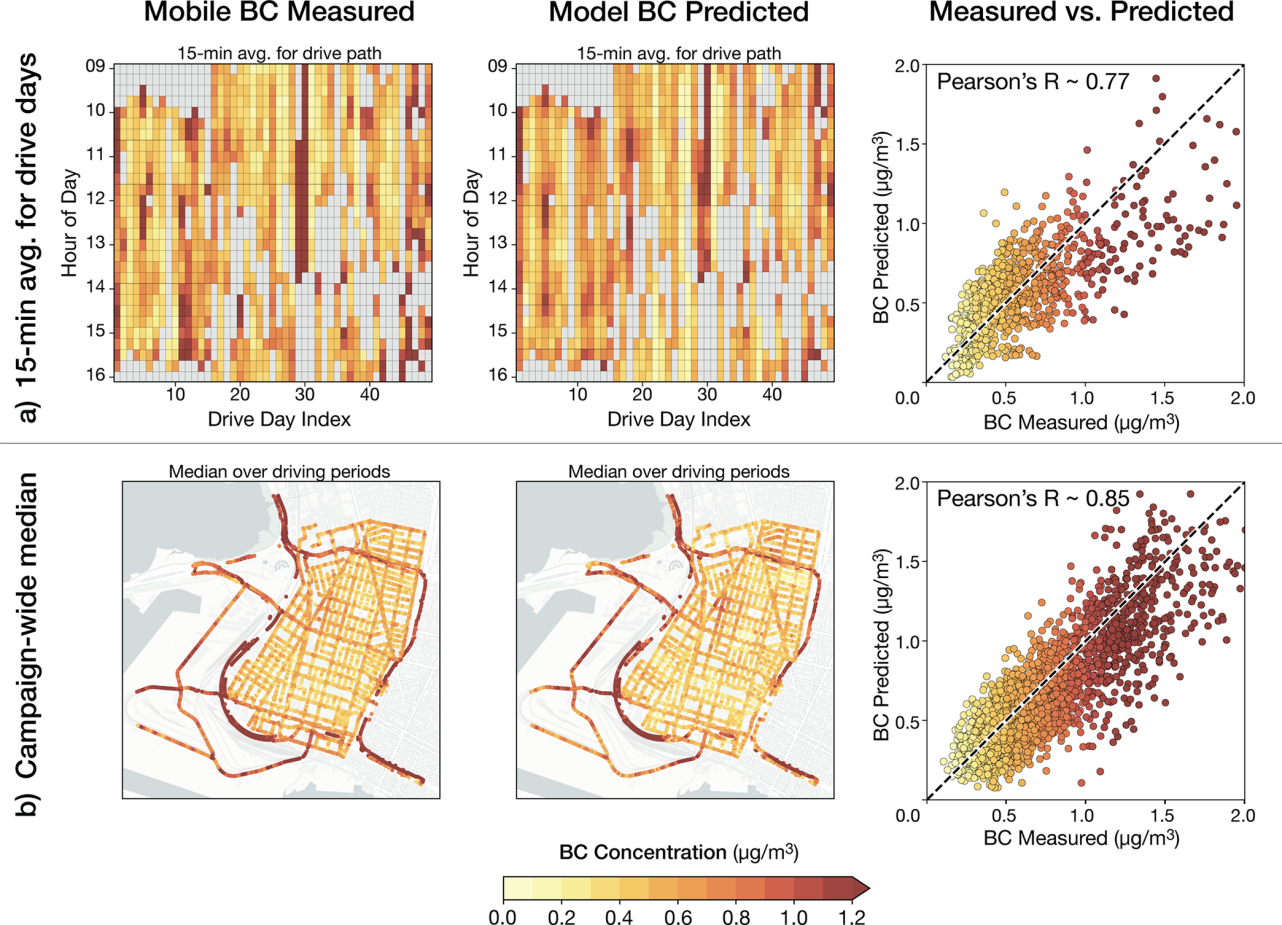
Evaluation of model performance across multiple spatiotemporal scales. (a) Comparison of the spatiotemporal averages along the sampled drive path for mobile measurements *(left)* and model predictions (*center*) for all days when mobile sampling occurred. Each data point and cell represents a 15 min “Lagrangian” spatiotemporal average along the vehicle drive path. (b) Temporal aggregation of time-resolved mobile measurements *(left)* and model predictions *(center)* to produce spatial maps of the campaign-integrated median BC concentrations. Note the high coherence between the spatial patterns of mobile measurements and model predictions.

**Figure 3:**
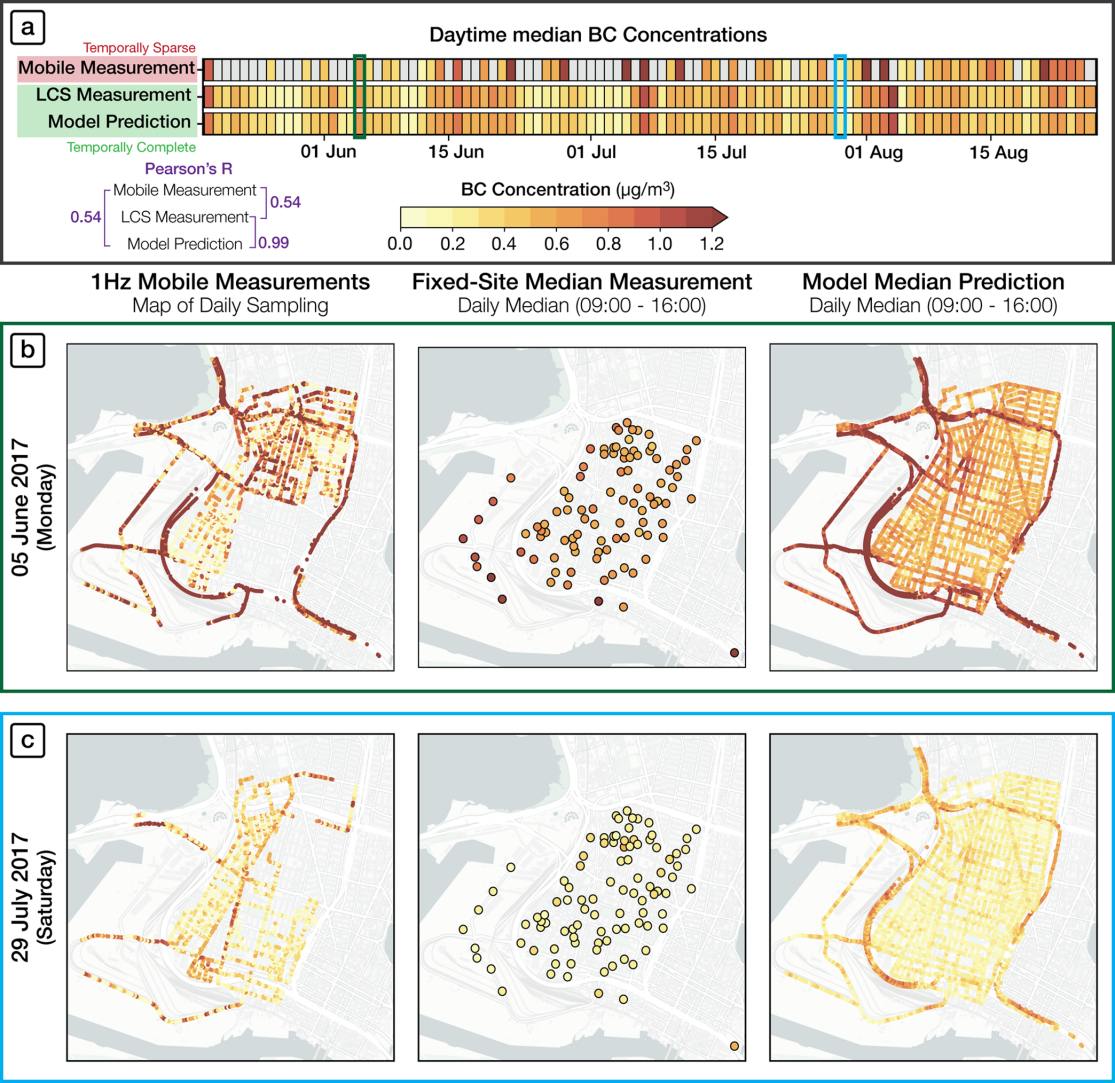
Temporal gap-filling. (a) Time-series heatmap shows median BC concentrations for daytime hours, comparing mobile measurements, fixed-site measurements, and model predictions. Note how the poor spatial and temporal representativeness of mobile measurements on any given day leads to a low correlation with measurements. Panels (b) and (c) show results for Monday June 5 and Saturday July 29. Maps from a single day of driving *(left)* have large spatial gaps and are not temporally representative. Fixed-site measurements *(center)* are temporally complete but spatially sparse. In contrast, predicted BC maps from the model *(right)* provide spatial completeness at any temporal scale. Here, we show daytime median BC predictions for two different days, revealing distinct spatial patterns that likely arise from differences in activity, emissions, and meteorology.

**Figure 4:**
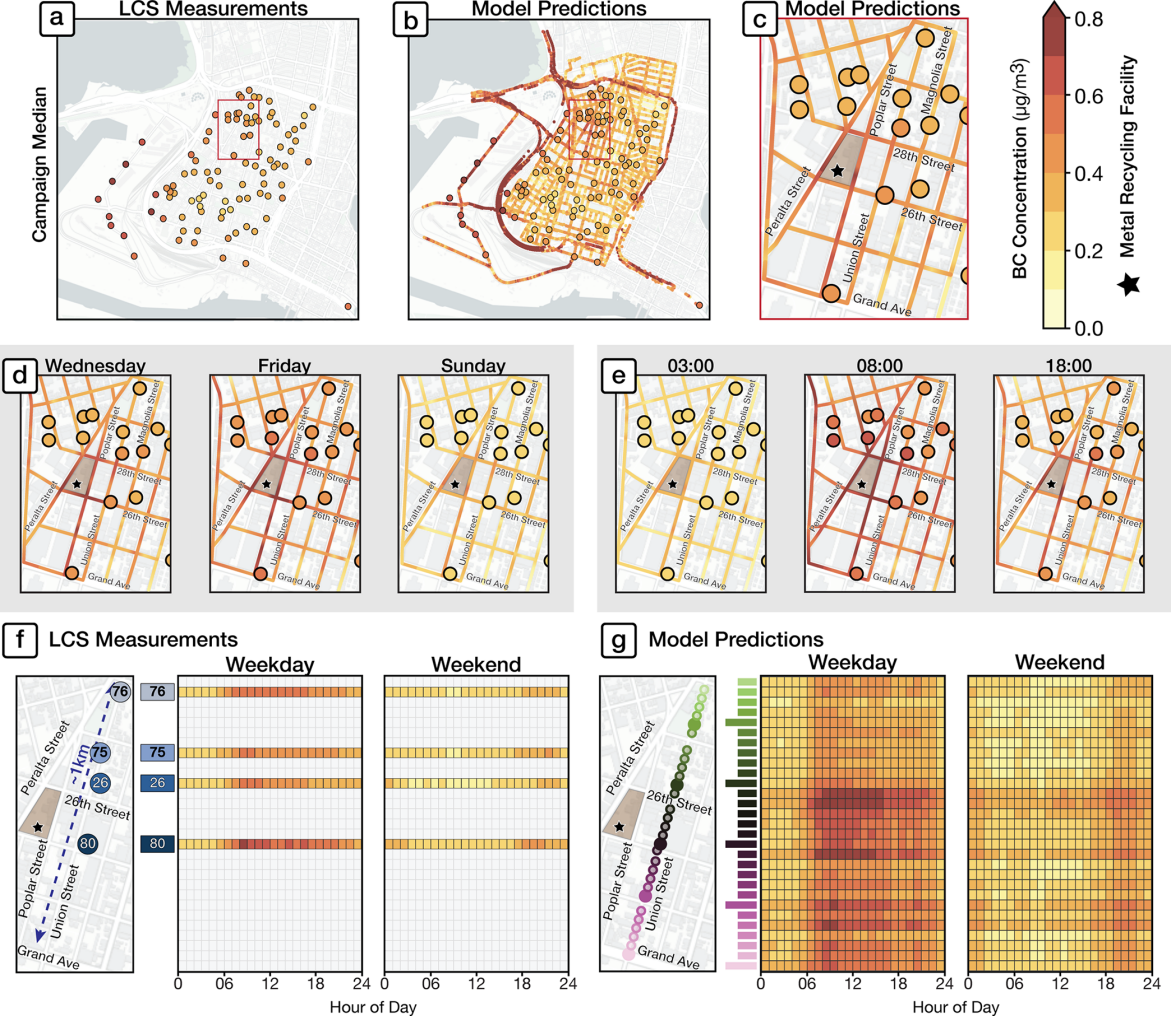
Spatial gap-filling of time-series data. (a) Campaign median of fixed-site BC measurements. (b) Median model predictions overlaid on the sensor network, revealing within-neighborhood variation. (c) Close-up view in a neighborhood surrounding a metal recycling cluster. This spatial gap-filling can be extended to distinct days of the week (d) or times of day (e), revealing localized pollution hotspots near emission sources that are not captured by the spatially sparser monitoring network. We contrast weekday and weekend diurnal time series along a single transect (Union Street) within the zoomed-in region. (f) Diurnal cycles for four closely spaced monitors within three city blocks (site IDs from Caubel et al.^28^), illustrating the presence of localized pollution sources. (g) Model predictions over the same ∼1 km transect, illustrating how the magnitude of BC and the corresponding diurnal signatures vary markedly among unobserved locations.

Here, we briefly describe the core model, which we present in Figures 2–4, as well as a set of five alternative models (see Table S1) developed to interrogate the robustness of our overall approach. The core model is developed using data from 97 fixed-site BC sensors, operating over the 100 days from May 19th to Aug 27th, 2017, and time-averaged multipollutant maps developed based on 49 days of available data from the two Google Street View cars operating in this time period. The factor analyses, core model, and all sensitivity test models were primarily developed using Python, leveraging multiple open-source libraries and packages (see SI Section S1.6 for details).

Sensitivity cases A and B aid in evaluating the out-of-sample prediction fidelity of our model. In sensitivity case A, we iteratively trained the model twice, each time using data from only one of the two Google Street View cars (and all of the data from the LCS sites), using the time-resolved and time-averaged data from the held-out car exclusively for model evaluation.^12^ In sensitivity case B, we trained the model on a randomly selected subset of 70% of the LCS sites (68) and iteratively refit the model 1000 times using different permutations of sites for the training. We then use the remaining 30% of fixed sites to test the out-of-sample prediction performance of the spatiotemporal model. In sensitivity case C, we explored how model performance depends on the number of overall LCS fixed sites by parametrically sampling and refitting the model 1000 times each for a random subset of 10 to 80 LCS sites. Since the model relies on “representative” time-averaged mobile maps^12^ rather than time-varying mobile measurements, in sensitivity case D, we explored the sensitivity of our results to the period of mobile monitoring data. To do so, we incorporated mobile monitoring from a random selection (repeated with 100 Monte Carlo iterations) of 49 drive days from other years and/or seasons collected during the time window between May 2015 and December 2017 but excluding the 100 day window of the LCS sampling campaign. In sensitivity case E, we systematically assessed how model performance is impacted by the number of pollutants (in addition to BC) measured using mobile monitoring (see Table S1).

## 3 Results and Discussion

Our spatiotemporal model estimates BC concentrations during 100 days at 15 min resolution for all ∼4300 30-m road segments in the ∼15 km^2^ West Oakland domain. We first describe our process for evaluating model performance and then discuss the key insights from the outputs.

### 3.1 Model Performance Evaluation

Because our model makes spatiotemporally complete estimates, whereas available observation data sets are sparser in space and/or time, careful consideration is needed to evaluate our predictions at an appropriate “apples-to-apples” spatiotemporal resolution. In addition to directly validating our core model against mobile and LCS data at multiple spatial, temporal, and spatiotemporal scales, we performed cross-validation analyses in sensitivity cases A and B to use fully independent data sets for model validation.

#### 3.1.1 Evaluation Using Mobile Monitoring Data

Because our model uses mobile monitoring data that has been extensively time-averaged, *time-varying* mobile BC measurements are useful for validation, especially for temporal performance (Figure 2). We compare our model output against mobile measurements in 15 min model timesteps using a “Lagrangian” approach, where we follow the vehicle’s sampling path. For each 15 min time window with available mobile data, we compare (i) the time average of the measured 1 Hz BC concentrations along the sampling route during this time interval and (ii) the spatial average of the 15 min-average model predictions for all road segments traversed by the vehicle (see SI Section S1.7 for details of our Lagrangian comparison method).

##### 3.1.1.1 15 min Spatiotemporal Averages

In Figure 2a, we compare 15 min spatiotemporal averages for mobile measurements (Figure 2a, left) and model predictions (Figure 2a, center). This evaluation spans all 49 days when mobile sampling took place during the 100 day observational period between 9 AM and 4 PM. We find a high correlation R = 0.77 between measured and predicted BC concentrations, even when assessed at a 15 min temporal resolution (Figure 2a, right). (Intriguingly, Figure S3 shows that that our model is also capable of reproducing spatial patterns *within* individual 15 min time intervals with good fidelity: median *R* = 0.52.) The model performs well in capturing 15 min-average concentrations with low to moderate BC concentrations (0.2–1.5 μg/m^3^) and tends to slightly underpredict the highest concentrations, with the peak absolute error typically falling within the order of approximately 25% (Figure S4). We attribute this in part to the model’s inherent limitations in accurately resolving sudden sharp peaks, a challenge shared by other spatiotemporal models. Figure S4a depicts residuals between measured and predicted spatiotemporal averages for all 15 min intervals across all drive days. The distribution of errors appears random, with no evident temporal bias (see Figure S4a).

We next investigate whether our model is capturing unique *spatiotemporal* features, rather than simply tracking the general temporal evolution of urban background concentrations, following the approach of Wai et al.^40^ We find that our core model has a substantially superior performance against mobile measurements than a time series of area-wide-average model predictions (*R* = 0.77 vs 0.54, see Figure S5), indicating that our model is capturing intricate spatiotemporal features, not just general temporal patterns that hold across the domain.

Finally, we iteratively held out one of the two cars’ monitoring from model building and then used those data for model testing (sensitivity case A, see Section 2.3 and Table S1). Our results show similar performance using one car exclusively for model validation, with strong spatiotemporal correlations of *R* = 0.73 and 0.80 (see Figure S6).

##### 3.1.1.2 Campaign-Integrated Spatial Patterns

Next, we assess the model’s ability to replicate observed spatial patterns for the 100 day average (Figure 2b). We compare the time-integrated spatial patterns (median-of-drive-pass-mean concentrations) that emerge from the full mobile monitoring campaign, comparing the aggregation of mobile measurements (Figure 2b, left) against the aggregation of time-resolved model predictions made along the Lagrangian trajectory of the mobile laboratories’ repeated visits to each 30 m road segment. This comparison shows an even stronger correlation, *R* = 0.85 (Figure 2b, right), with no systematic spatial pattern of residuals (Figure S4b). This investigation highlights that the spatial performance of the model improves somewhat when averaging over a longer period.

#### 3.1.2 Evaluation Using Fixed-Site LCS Data

To assess the skill of our model at predicting time-series data at fixed sites, we repeatedly used the 30% holdout scheme we described in sensitivity case B to make out-of-sample predictions at LCS sites excluded from model development (see Section 2.3, Table S1, and Figure S7a). The results indicate a very high correlation of predicted and measured BC time series (for the median out-of-sample site, the temporal R = 0.95, 10th to 90th percentile range of 0.80–0.97) at the LCS network sites as shown in Figures S7 and S8. The normalized mean bias (NMB) is approximately −5%, while the normalized mean absolute error (NMAE) and normalized root-mean-squared error (NRMSE) are 20 and 25%, respectively. Thus, on average, the model exhibits a slight tendency to underpredict the observed concentrations. It is interesting to contrast our results with those from the spatiotemporal model of Wai et al.,^40^ who report a lower mean temporal *R*^2^ value of 0.60 (compared to a Pearson *R*^2^ of ∼0.90 here) when predicting LCS measurements in a similar leave-site-out evaluation. We attribute the improvement in temporal performance in part to the additional spatial information that mobile monitoring data contributes to our spatiotemporal model.

Considering that mobile monitoring was predominantly conducted on weekdays from 9 AM to 4 PM, we also assessed whether this limited temporal coverage impacts model performance during time periods when only LCS measurements are available. To do so, we segmented the model output by weekday/weekend and daytime/nighttime categories for each of the leave-site-out cross-validation trials. Figure S7b provides an overview of these results, demonstrating consistent model performance across all temporal subset conditions. This result is noteworthy as it underscores that the model’s performance remains relatively unaffected by the absence of mobile measurements during specific days or times, suggesting that the continuous temporal coverage provided by the LCS enables the model to dynamically adjust the weighting of the spatially coherent patterns derived from mobile monitoring data. As a result, the model can identify spatial patterns that might be missed by LCS sites.

### 3.2 Model Application: Filling in Spatiotemporal Gaps

Next, we explore how the spatiotemporal model can provide new insights by filling monitoring gaps in space and time.

#### 3.2.1 Temporal Gap-Filling

We start with an illustration (Figure 3), which depicts how temporally complete model predictions can offer new insights into the complete spatial patterns of BC at finer time resolution than would be possible with the temporally incomplete mobile monitoring data. Figure 3a depicts the daily time series of spatial averages of daytime (9:00–16:00) median BC concentrations as observed by mobile measurements, LCS measurements, and model predictions over the 100 day study period. We find that the time series of daily spatial averages of our spatiotemporal model closely reproduces that of the LCS sensors (*R* = 0.99). In contrast, we find a lower temporal correlation (*R* = 0.54) between the spatial averages of the daily mobile measurements and our spatiotemporal model. This result makes sense. Within just 1 day of driving, mobile monitoring does not generally capture spatially representative concentration patterns, whereas a spatiotemporally complete model can represent fine-scale spatial patterns for a single day. Moreover, temporally complete model predictions can help address notable limitations of mobile monitoring, including subtle but irreducible temporal sampling biases and the emphasis on daytime sampling.

Next, we contrast daily median spatial patterns for two illustrative days during the campaign: Monday, June 5th, and Saturday, July 29th, 2017 (Figure 3b–c). For mobile measurements, maps derived from a single drive pass are inherently spatially complete and temporally unrepresentative (Figure 3b–c, *left*). In contrast, fixed-site measurements (Figure 3b–c, *center*) and model predictions (Figure 3b–c, *right*) are temporally complete and thus are representative of spatial variability for a given day. Our daily model predictions are well correlated with LCS observations for these 2 days (spatial *R* = 0.8 and 0.75, respectively; see Figure S9).

Relative to the LCS network, a key advantage of the model is that it can reveal distinct spatial patterns linked to activity that differ from day to day, including sharp concentration gradients near highways, industrial sites, or truck routes converging to the port. Many of these spatial features are in areas overlooked by the sparser LCS network. The time-resolved (i.e., daily) spatial patterns from the model output (Figure 3b–c, *right*) can deviate substantially from the campaign-averaged map of mobile measurement (cf. Figure 2b, *left*), revealing intermittent spatial features that are the signature of many of the BC sources in the study domain.

#### 3.2.2 Spatial Gap-filling of Time-Series Data

We next consider how the spatiotemporal model can fill spatial monitoring gaps. We demonstrate that our spatiotemporal model reveals how the temporal variation in BC levels can be quite distinct over even short spatial distances.

To motivate this discussion, we first compare domain-wide maps of the campaign medians of the fixed-site LCS measurements (Figure 4a), and the spatiotemporal model overlaid on the LCS measurements (Figure 4b). Figure 4c provides a zoomed-in view of a single neighborhood containing both residences and multiple industrial facilities, where model predictions exhibit fine-scale spatial variability not captured by the sparser fixed-site sensors. Considering distinct days of week and times of day, Figures 4d-e illustrate that while the LCS sites capture the overall contrast, the finer-scale spatial variation of the model reveals more about the locations of specific activities. Model predictions are overlaid onto LCS sites, revealing the spatial variability captured versus missed across different days of the week or times of the day. The pronounced contrast in fine-scale spatial features, particularly comparing Friday to Sunday (weekday/weekend) and 3:00 am to 8:00 am (night/day), illustrates how spatial features correspond with the operating hours of major emissions sources in the neighborhood.

To further illustrate the model’s proficiency in capturing fine-scale spatial patterns over time, we investigate the fine-scale spatial variability of the time series predicted by the model. We examine how the diurnal variation of BC varies along a ∼ 1 km spatial transect defined by a specific road, Union Street (Figure S10). Figure 4f shows heatmaps of the *measured* diurnal cycles for four closely spaced LCS sites within three city blocks along Union Street. The diurnal patterns vary across the four sites, revealing localized pollution hotspots near facilities such as a metal recycling plant and a moving company.^28^ We find that the *model* is capable not only of reproducing similar diurnal profiles (Figure S11) at these four locations (Figure 4g) but also reveals sharp variation in the diurnal profiles at *unobserved* locations along the Union Street transect. These nuanced diurnal signatures over short distances correspond to localized differences in the microenvironment in this mixed neighborhood, including major roadways, areas near industrial sites, and residential zones. Imagery in Figure S10 provides further context to these findings. This vignette within one heterogeneous neighborhood shows how a spatiotemporally complete model can fill measurement gaps to identify intricate spatial patterns in BC concentrations.

### 3.3 Limitations and Design Considerations for Future Campaigns

In the present study, we were able to leverage an unusually rich data set of mobile and fixed-site monitoring. We conducted a series of data experiments to explore the potential of our method to work with monitoring data that is more limited along multiple distinct axes: fewer fixed sensors, less temporally representative mobile monitoring, or fewer pollutants.

In sensitivity case C (see Section 2.3 and Table S1), we explore how model performance varies as a function of fixed sites used to construct the model. We find sharply diminishing returns to model performance after including more than 40 *randomly* selected sites out of the existing 100 sites (Figure S12). If we had sufficient information (e.g., from an initial deployment of many LCS) to place sensors *optimally* rather than randomly, fewer sensors would be required (see SI Section S1.8).^43^ In Figure S13, we illustrate results from an optimal sensor placement algorithm,^45^ which suggests that as few as 20 well-placed sensors would enable us to reproduce the performance of our core model. As detailed in Section S1.8, the optimal sensor placement algorithm is designed to maximize the spatiotemporal variability captured for a given number of sites. Although the algorithm is purely data-driven, the 20 sites identified as optimal in Figure S13e are located near industrial facilities, designated truck routes, freeways, the port, and railyards—areas where high spatiotemporal variability in BC emissions is expected.

Likewise, it may not always be feasible to simultaneously collect mobile and fixed-site measurements. However, since our model only requires time-averaged maps from mobile measurements, we used sensitivity case D to assess whether mobile measurement maps derived from a period outside the fixed-site measurement’s time frame could still yield viable results (see Section 2.3 and Table S1). As shown in Figure S14, we found our model’s spatiotemporal performance declined modestly (median *R =* 0.67 vs 0.77) when trained on mobile monitoring data outside of the 100 day study period.

Another consideration is the number of unique pollutants measured by multipollutant mobile monitoring. In sensitivity case E, we systematically considered models developed with combinations of BC and fewer than the 9 total species measured by our mobile monitoring. As we increased the number of species incorporated from 4 to 10 (Figure S15), we found meaningful improvement in *R* (from ∼0.6 to 0.77) and other measures of model performance. We attribute this improvement to the broader range of pollutants, along with their shared variance, which enables the model to better identify pollutant-invariant patterns and thereby more effectively fill spatiotemporal gaps. However, with the available evidence, it is difficult to reliably isolate the unique influence of a specific pollutant on model performance since pollutants have correlated spatial patterns (Figure S2) that arise because of common sources and meteorology. Rather, Figure S15 suggests that the model is more sensitive to the number of pollutants included than to which pollutants are included, implying that our factor analysis benefits principally from having a large set of pollutants, each with somewhat unique spatial patterns. We do not see clear evidence of diminishing returns to adding additional pollutants to the mobile suite from *n =* 4 to 10.

It remains an open question whether this modeling approach would perform well for reactive or secondary air pollutants. Likewise, it would be useful to confirm our findings for BC in other settings with different sources and meteorology. Replicating this study’s resource- and labor-intensive level of monitoring may not be feasible everywhere. However, our sensitivity analyses suggest that this modeling technique is viable even with considerably fewer mobile or fixed-site measurements and thus would provide useful information in filling in spatiotemporal gaps from monitoring. Nonetheless, as a data-driven technique, a key limitation is that our approach is not capable of predicting concentrations for time periods or spatial domains that altogether lack measurements. Moreover, in contrast to physics-based models (e.g., dispersion or chemical-transport models), our model is not capable of simulating the impact of emissions changes or other interventions.

## 4 Implications and Future Work

Finely resolved measurements of air pollution in space and time are increasingly needed. This study demonstrates the benefits of combining two disparate monitoring strategies rather than relying on low-cost sensors or mobile monitoring in isolation for capturing the spatiotemporal variation of a primary pollutant (e.g., BC) within a complex urban environment. The novel spatiotemporal model presented here integrates these measurements effectively, revealing fine-scale features in space and time that would be overlooked if considering one monitoring approach individually. A unique aspect of this approach is in using spatial patterns recurring over multiple pollutants collectively to fill spatial gaps observed for a single pollutant over time. Future endeavors can also explore extending the model’s applicability to other pollutants, particularly reactive ones, and to regions with different meteorological regimes.

Our modeling approach holds potential for broader applications. Future investigations could assess the impact of human mobility on pollutant exposures using finely resolved spatiotemporal models.^54^ Additionally, the spatiotemporal completeness provided by our model ensures spatial and temporal representativeness when aggregating the output at different scales. This feature may be particularly valuable for epidemiological studies, which often require processed data products like exposure estimates at residential addresses or census block level, either at a time-resolved or time-averaged scale. The fine resolution and completeness of our model output ensure the representativeness of these aggregates in such scenarios. In the future, this modeling approach may prove useful in expanding the capabilities of emerging hyperlocal air pollution observation systems. If applied over periods of time with changing emissions (e.g., in response to control policies, changes in traffic, or the addition of new sources), this method may aid accountability studies in identifying zones of changing emissions and exposure impact.^55^

## Supporting Information Available

Additional methodological details, sensitivity analyses, and supporting information figures (PDF)
