# Supplementary material for: Integrating Mobile and Fixed-Site Black Carbon Measurements to Bridge Spatiotemporal Gaps in Urban Air Quality
Source: Environ Sci Technol. 2024 Jul 1;58(28):12563–74. doi: 10.1021/acs.est.3c10829 (PMC11256762; doi:10.1021/acs.est.3c10829)
Supplement: Supplementary file 1 — es3c10829_si_001.pdf [file es3c10829_si_001.pdf]

Supporting Information

Integrating Mobile and Fixed-Site Black Carbon Measurements to Bridge Spatiotemporal Gaps in Urban Air Quality

Chirag Manchanda,[a] Robert A. Harley,[a] Julian D. Marshall,[b] Alexander J. Turner,[c] Joshua S. Apte [a,d,*]

[a] Department of Civil and Environmental Engineering, University of California, Berkeley, CA 94720, USA

[b] Department of Civil and Environmental Engineering, University of Washington, Seattle, WA 98195, USA

[c] Department of Atmospheric Sciences, University of Washington, Seattle, WA 98195, USA

[d] School of Public Health, University of California, Berkeley, CA 94720, USA

* Corresponding author. Email apte@berkeley.edu

This PDF file includes 30 pages containing:

1. Materials and Methods
2. SI References
3. Table S1
4. Figures S1 to S15

S1. Materials and Methods

S1.1 Optimization of Averaging Timescale for Fixed-Site Measurements

The native time resolution of the LCS network deployed by Caubel et al.[1] was 0.5 Hz. However, due to instrument noise considerations, Caubel et al. [1] opted to average the data down to a 1-minute time base. Building on this, Chambliss et al.[2] carried out a comparative examination of black carbon (BC) concentrations utilizing both fixed-site LCS and mobile measurements. They determined that averaging fixed-site measurements to a time resolution of 10-15 minutes struck a balance between preserving spatial heterogeneity captured by the LCS network and mitigating instrument noise and BC instrumentation disparities between the mobile and fixed platforms. Here, we explore the variability of the signal-to-noise ratio (SNR) associated with fixed-site measurements across averaging time periods ranging from 1 minute to 120 minutes.

It is essential to underscore that the selection of an appropriate averaging timescale relies on optimization rather than adherence to absolute metrics. Here, we ascertain the optimal averaging time scale by identifying the knee/elbow point along the curve depicting the variation of the signal-to-noise ratio (SNR) with different averaging time periods. Utilizing the `kneed` Python package,[3] which assesses the degree of curvature at discrete points on the normalized curve, we determined an optimal value for the averaging timescale. Figure S1a illustrates the relationship between SNR and increasing averaging time scale. The knee point on this curve occurs at 15 minutes, consistent with the findings reported by Chambliss et al.[2] Consequently, we choose to average the fixed-site data over 15-minute time intervals.

S1.2 Instrumentation

The mobile measurement data utilized in this study was derived from a subset of a broader ongoing mobile monitoring initiative, employing two custom-equipped Google Street View cars outfitted with the Aclima Ei measurement and data acquisition platform (Aclima, Inc., San Francisco, CA). As detailed in section 2.1, the current configuration was designed to monitor BC, NO, NO_2, ultrafine particles [UFP] and 6 size-resolved particle concentrations bins from 0.3 µm – 10 µm). Both the cars employed with an identical set of fast-response (1-Hz) laboratory-grade analyzers. NO concentrations were measured using chemiluminescence (Model CLD64, EcoPhysics AG, Switzerland). NO_2 was measured using a 450 nm cavity-attenuation phase-shift spectroscope (Model T500U, Teledyne Inc., San Diego, CA). UFP concentrations were measured using a water-based condensation particle counter with an effective minimum detection size of particle diameter exceeding 2.5 nm (Model 3788, TSI Inc., Shoreview, MN). Size-resolved particle number concentrations were measured using a portable optical particle counter (Model GT-526S, Met One Instruments, Grants Pass, OR).

Black carbon measurements on the mobile platform were conducted using a photoacoustic extinctiometer (PAX, Droplet Measurement Technologies, Longmont, CO),[4] whereas the fixed-site BC measurements relied on custom low-cost Aerosol Black Carbon Detector (ABCD).[5] Further details regarding the sampling inlet design, data acquisition and management system, as well as the routine calibration, quality assurance and quality control procedures implemented, can be found in Apte et. al.[6] and Caubel et. al.[1]

S1.3 Compressive Sensing viewpoint of the spatiotemporal modeling framework.

Naturally evolving systems often exhibit multidimensional characteristics with repeating patterns or coherent structures across one or more dimensions e.g. space, time, or chemical composition. Compressive sensing leverages these coherent structures or basis functions to represent the entire system dynamics with a sparse set of observations. Compressive sensing has found wide applications in various domains, including video/image compression, audio-visual processing, and signal processing.[7–9] Here, we describe how prior literature has utilized this technique in the context of air quality monitoring and explains how the present study draws inspiration from and extends the principles of compressive sensing.

S1.3.1 Tailored Compressive Sensing and Optimal Sensor Placement.

Conventional applications of compressive sensing commonly rely on universal or generic basis functions, such as the Fourier basis, for broader signal reconstruction.[9] However, recent studies have investigated the adoption of basis functions tailored to specific data, often employing matrix decomposition strategies to derive these tailored basis functions.[7] In the context of air quality applications, these tailored compressive sensing techniques have been employed to inform optimal sensor placement strategies.[10,11] The primary focus of these studies revolves around maximizing information capture with a reduced number of measurements, capitalizing on the insights offered by the tailored basis functions.

As elaborated in Section 2.2, let matrix $\mathbf{X}_{\boldsymbol{F}} \in \mathrm{R}^{\mathrm{s} \times \mathrm{t}}$ represent measurements of a pollutant at s sites and t timesteps. Following equation 2 in Section 2.2, this matrix can be decomposed into $\mathbf{W}_{\boldsymbol{S}}$, representing the time-invariant spatial patterns or the tailored basis in this case, and the corresponding characteristic time signals $\mathbf{H}_{\boldsymbol{T}}$ (refer to Section 2.2 for details):

$$\mathbf{X}_{\boldsymbol{F}} = \mathbf{W}_{\boldsymbol{S}}\mathbf{H}_{\boldsymbol{T}} \qquad \mathbf{W}_{\boldsymbol{S}} \in \mathrm{R}^{\mathrm{s} \times \mathrm{q}}; \mathbf{H}_{\boldsymbol{T}} \in \mathrm{R}^{\mathrm{q} \times \mathrm{t}} \qquad (S1)$$

Such that a single vector column $x_F \in \mathrm{R}^{\mathrm{s} \times 1}$ can be expressed as:

$$x_F = \mathbf{W}_{\boldsymbol{S}} h_T \qquad \mathbf{W}_{\boldsymbol{S}} \in \mathrm{R}^{\mathrm{s} \times \mathrm{q}}; h_T \in \mathrm{R}^{\mathrm{q} \times 1} \qquad (S2)$$

Here, x_F represents the measurements across all s sites at a single timestep, now expressed in terms of the tailored basis $\mathbf{W}_{\boldsymbol{S}}$ and corresponding factor loadings or weights h_T. In simpler terms, this implies that measurements at any timestep can be redefined as a weighted (h_T) sum of some spatial patterns ($\mathbf{W}_{\boldsymbol{S}}$) that persist across time. Extending this to measurements across all timesteps ($\mathbf{X}_{\boldsymbol{F}}$), these factor loadings translate to the characteristic time signals ($\mathbf{H}_{\boldsymbol{T}}$) corresponding to the basis ($\mathbf{W}_{\boldsymbol{S}}$).

Now, from an optimal sensor placement viewpoint, measurements at all these sites are not equally important. Hence, an optimal sensor placement strategy focuses on designing a binary sampling matrix $\emptyset_{\boldsymbol{c}}$ to select a compressed set of measurements $\mathbf{X}_{\boldsymbol{CF}}$ at r of the original s sites:

$$\mathbf{X}_{\boldsymbol{CF}} = \emptyset_c \mathbf{X}_{\boldsymbol{F}} \qquad \mathbf{X}_{\boldsymbol{CF}} \in \mathrm{R}^{\mathrm{r} \times \mathrm{t}}; \emptyset_{\boldsymbol{c}} \in \mathrm{R}^{\mathrm{r} \times \mathrm{s}} \qquad (S3)$$

Given $\mathbf{W}_{\boldsymbol{S}}$, $\emptyset_{\boldsymbol{c}}$ is selected in a way that $\mathbf{X}_{\boldsymbol{CF}}$ alongside $\mathbf{W}_{\boldsymbol{S}}$, can be used to derive an optimal estimate $\mathbf{X}_{\boldsymbol{F}}'$ of the original set of measurements $\mathbf{X}_{\boldsymbol{F}}$. Given $\mathbf{W}_{\boldsymbol{S}}$, $\emptyset_{\boldsymbol{c}}$ and $\mathbf{X}_{\boldsymbol{CF}}$ using equations (S1) and (S2) we can write:

$$\mathbf{H}_{\boldsymbol{T}}' = (\emptyset_c \mathbf{W}_{\boldsymbol{S}})^{\dagger} \mathbf{X}_{\boldsymbol{CF}} \qquad (S4)$$

where †, represents the Moore Penrose pseudo inverse. Finally, we can express:

$$\mathbf{X}_{\boldsymbol{F}}' = \mathbf{W}_{\boldsymbol{S}} \mathbf{H}_{\boldsymbol{T}}' \qquad (S5)$$

S1.3.2 Spatiotemporal model and its departure from conventional tailored compressive sensing

As discussed in section S1.3.1, optimal sensor placement strategies are focused on selecting a compressed set of measurements $\mathbf{X}_{\boldsymbol{CF}} \in \mathrm{R}^{\mathrm{r} \times \mathrm{t}}$ from a given set of fixed-site measurements $\mathbf{X}_{\boldsymbol{F}} \in \mathrm{R}^{\mathrm{s} \times \mathrm{t}}$, across fewer ($r <$ s) optimally placed sites. However, the spatiotemporal modeling

framework, as detailed in section 2.2, takes a different perspective. Here, we interpret the fixed-site measurements $\mathbf{X}_F$ as a compressed set of measurements within a hypothetical, much denser observation network, such that:

$$\mathbf{X}_F = \emptyset\mathbf{X}_{BC} \qquad \emptyset \in \mathrm{R}^{s \times l};\ \mathbf{X}_{BC} \in \mathrm{R}^{l \times t} \qquad (S6)$$

Similar to equation S3, the binary sampling matrix $\emptyset \in \mathrm{R}^{s \times l}$ selects s fixed-site measurements from the much denser set of hypothetical fixed-site measurements $\mathbf{X}_{BC} \in \mathrm{R}^{l \times t}$, such that ($l \gg$ s). By utilizing equation S1, $\mathbf{X}_F = \mathbf{W}_S\mathbf{H}_T$, we express

$$\mathbf{W}_S\mathbf{H}_T = \emptyset\mathbf{X}_{BC} \qquad (S7)$$

If we can estimate a spatially augmented version of the basis $\mathbf{W}_S$, denoted as $\widetilde{\mathbf{W}_S} \in \mathrm{R}^{l \times q}$, such that:

$$\mathbf{W}_S = \emptyset\widetilde{\mathbf{W}_S} \qquad (S8)$$

Then, we can derive estimates of the pollutant measurements corresponding to the much denser hypothetical fixed-site network:

$$\mathbf{X}_{BC} = \widetilde{\mathbf{W}_S}\mathbf{H}_T \qquad \widetilde{\mathbf{W}_S} \in \mathrm{R}^{l \times q};\ \mathbf{H}_T \in \mathrm{R}^{q \times t} \qquad (S9)$$

The spatiotemporal model in this study estimates the spatially augmented basis $\widetilde{\mathbf{W}_S}$, by combining time-averaged multi-pollutant mobile measurements with $\mathbf{W}_S$ and $\emptyset$. As discussed in detail in section 2.2 and derived from equations (1-3), *pollutant–invariant* patterns $\mathbf{H}_L{}^{\boldsymbol{T}} \in \mathrm{R}^{l\times k}$derived from mobile measurements, can be reprojected on *time–invariant* patterns $\mathbf{W}_S \in \mathrm{R}^{s \times q}$ from fixed-site measurements using coefficient matrix $\mathbf{C} \in \mathrm{R}^{k\times q}$, such that:

$$\mathbf{W}_S \approx \boldsymbol{\emptyset}\mathbf{H}_L{}^{\boldsymbol{T}}\boldsymbol{C} \qquad (S10)$$

Comparing equations, (S8) and (S10)

$$\widetilde{\mathbf{W}_S} = \mathbf{H}_L{}^{\boldsymbol{T}}\boldsymbol{C} \qquad (S11)$$

Thus, combining (S9) and (S11):

$$\mathbf{X}_{BC} = \mathbf{H}_{L}{}^{T} \boldsymbol{C}\, \mathbf{H}_{T} \quad (S12)$$

S1.4 Cross-correlation and NMF reconstruction efficiency among time averaged multi-pollutant maps

In Section 2.2, the discussion revolves around the hypothesis that common emission sources and meteorological conditions exert influence on the spatial patterns of various pollutants across multiple time scales. It suggests that spatial features observed across multiple pollutants can assist in bridging gaps between spatial features across time. Figure S2(a) demonstrates the cross-correlations between different pollutant concentration maps derived from routine mobile monitoring, which serve as the *pollutant-location* system ($\mathbf{X}_{M}$) input to the model in Section 2.2. While there is moderate to high correlation among multiple pollutants (with R ranging from 0.5 to close to 1), the applicability of the model presented in Section 2.2 relies on whether the measured pollutants have enough shared covariance for coherent patterns to be extracted.

From a factor analysis standpoint, for the pth pollutant vector in $\mathbf{X}_{M}$, say $x_{1\times l}$ the variance exhibited by $x_{1\times l}$, can be partitioned into *common variance* (*communality*) and unique variance (including error):

$$Var(x_{1\times l}) = Communality + Unique\ Variance \quad (S13)$$

Factorization techniques like NMF as employed in section 2.2 aim to decompose $\mathbf{X}_{M}$ into a pollutant subspace $\mathbf{W}_{P}$ and a location subspace $\mathbf{H}_{L}$, using NMF as follows:

$$\mathbf{X}_{M} = \mathbf{W}_{P}\mathbf{H}_{L} \qquad \mathbf{W}_{P} \in \mathrm{R}^{\mathrm{p\times k}}; \mathbf{H}_{L} \in \mathrm{R}^{\mathrm{k\times l}} \quad (S14)$$

So, now for the pth pollutant vector $x_{1\times l}$

$$x_{1\times l} = \mathrm{w}_{1\times k}\mathrm{h}_{k\times l} + error \quad (S15)$$

NMF relies on communality to explain the variance of the input variables and cannot account for the unique variance, which remains as error when trying to reconstruct the original input variables from the NMF-derived decomposition.

For the spatiotemporal model to function effectively, the set of measured pollutants should share enough variance so that most of the spatial features can be reconstructed from the NMF-derived coherent patterns. Figure S2(b) illustrates the correlation between the NMF-reconstructed multipollutant concentration maps (derived using equation 1) and the original input. It demonstrates that Pearson's R is 0.995 for BC (our pollutant of interest), indicating that almost all the spatial variance exhibited by BC is captured by the 5 pollutant–invariant spatial patterns extracted by equation 1, making BC a suitable application for the model. Likewise, all other pollutants also demonstrate high correlation coefficients between input and reconstructed patterns, highlighting the significant impact of shared emissions and meteorological conditions on the variation of multiple pollutants for the geographical region and time period of our study. However, relative to the other pollutants, UFP appears to have the highest fraction of unique variance (due to its lowest relative correlation), suggesting the influence of unique sources impacting UFP not shared by the other pollutants under consideration.

S1.5 Determination of Optimal Rank for Matrix Decomposition

The spatiotemporal model proposed in this study relies on extracting coherent spatial patterns from both the *pollutant-location* and *location-time* systems (refer to section 2.2 for details). Non-negative matrix factorization (NMF) is employed to decompose the *pollutant-location* and *location-time* matrices and extract these patterns. However, NMF, like any matrix factorization method, requires the user to input the rank for the decomposed matrices during factorization. To ascertain the optimal rank for factorization, we track the normalized residual sum of squares,

normalized through min-max scaling, across various iterations involving different matrix ranks. The optimal rank is determined by identifying the knee/elbow point along the curve, representing a significant transition in the residual sum of squares.

For the *pollutant-location* system ($\mathbf{X}_M$), we systematically vary the number of factors from 2 to 10. Figure S1b illustrates how the residual sum of squares for this decomposition decreases as the number of factors increases. The knee point on this curve is observed at 5, indicating that 5 is the optimal rank for decomposing the $\mathbf{X}_M$ matrix. Similarly, for the *location-time* system ($\mathbf{X}_F$), representing the fixed-site BC measurements matrix, the number of factors is varied from 4 to 64 with a factor of 2 (i.e., 4, 8, 16, 32, and 64). Figure S1c demonstrates the variability of the residual sum of squares as the number of factors increases. The knee point on this curve is found to be 16, signifying that 16 is the optimal rank for factorizing the $\mathbf{X}_F$ matrix.

To further ensure the optimality of the chosen decomposition rank for both the matrices ($\mathbf{X}_M$ **and** $\mathbf{X}_F$), we employed a bootstrap random resampling strategy, as detailed by Brown et al.[12] for the rank indicated by the knee point along with the neighboring ranks i.e., 4, 5 & 6 for the *pollutant-location* system ($\mathbf{X}_M$), and 8, 16 & 32 for the *location-time matrix* ($\mathbf{X}_F$). The bootstrap resampling is a common strategy employed for testing optimal ranks for matrix factorization.[12] The bootstrap procedure involves randomly selecting non-overlapping blocks of species measurements (along locations for $\mathbf{X}_M$ and along timesteps for $\mathbf{X}_F$) from the input dataset, creating a new input matrix with an equivalent number of samples as the original. This new matrix is then subjected to NMF again with the decomposition ranks to be tested. Subsequently, the bootstrapped NMF factors are mapped to the primary factors. The bootstrapped factors are assigned to corresponding base factors based on the highest uncentered correlation values above a user-specified threshold (Pearson R of 0.8 for our study). Any bootstrap factor failing to meet this

correlation threshold with any base factor is considered "unmapped." This analysis provides a means to assess the uncertainty associated with the solution and the resolved factors for the chosen decomposition rank.

In our study, each of the six cases (3 for $\mathbf{X}_{\boldsymbol{M}}$ and 3 for $\mathbf{X}_{\boldsymbol{F}}$), underwent 1000 bootstrap runs each, with a threshold correlation of 0.8. For both $\mathbf{X}_{\boldsymbol{M}}$ and $\mathbf{X}_{\boldsymbol{F}}$, the rank identified as optimal by the knee-point evaluation (5 for $\mathbf{X}_{\boldsymbol{M}}$ and 16 for $\mathbf{X}_{\boldsymbol{F}}$) was confirmed as an optimal solution by the bootstrap analysis. Moreover, in more than 900 runs, there were no unmapped factors (938 for $\mathbf{X}_{\boldsymbol{M}}$ and 903 for $\mathbf{X}_{\boldsymbol{F}}$), while the number of unmapped factors was higher for other tested ranks for both matrices, indicating potential underfitting or overfitting.

S1.6 Description of Python packages used for model development.

Our spatiotemporal model was developed primarily using Python, with the aid of various open-source libraries and packages for data analysis. Key Python packages utilized in our study include:

- `nimfa`[13] library: Used for Non-negative Matrix Factorization (NMF).
- `pysensors`[7] library: Used for sensor placement optimization.
- `xarray`[14] and `pandas`[15]: Employed for data organization and operations.
- `geopandas`[16]: Facilitated geospatial operations.
- `scipy`[17]: Applied for data analytics tasks.
- `cmcrameri`[18] package: Integrated for color vision deficiency-friendly color maps.

S1.7 Spatiotemporal averaging for temporal alignment of model output and mobile measurements

The mobile measurements utilized in this study have a native resolution of 1 Hz. These instantaneous measurements are subsequently subjected to averaging for each 30-meter road segment covered by the vehicle, consolidating around 2-10 instantaneous measurements at typical

speeds (refer to Section 2.1 for details). In contrast, the model predicts the average concentration for every 30-meter road segment across the spatial domain during a given 15-minute period, leading to a temporal misalignment between model predictions and mobile measurements. To reconcile this misalignment in both space and time, we employ a 'Lagrangian' spatiotemporal averaging approach, tracking the instantaneous trajectory of each mobile monitoring vehicle during discrete 15-minute intervals.

In Figure S3, we present a comparison between the mobile measurements obtained during a single indicative 15-minute interval during the campaign (Figure S3a) and 15-minute average (for the same time interval) model predictions for the corresponding road segments (Figure S3b). Despite disparities in temporal resolution, Figure S3c reveals a Pearson's correlation coefficient of $R = 0.67$ between the two datasets for this example. Similar comparisons can be extended to 1475 distinct 15-minute periods across the 49 drive days with mobile measurements. The distribution of R for these periods is depicted in Figure S3d, ranging between 0.28 and 0.75 (10th and 90th percentiles, respectively), with a median value of 0.52.

For a more direct comparison, we further condense the mobile measurements for each of these distinct 15-minute intervals into single data points. These condensed measurements are then compared against the spatial average of the model predictions (inherently a 15-minute time average) over corresponding road segments for each 15-minute interval (see Section 3.1.1 and Figure 2 for details). For the example case presented in Figure S3, this translates to averaging the measurements presented in Figure S3a into a single point and comparing them to the subset of model predictions presented in Figure S3b, condensed to one point.

It is crucial to note that while this averaging approach aligns both measurements and model predictions to represent the same set of points spatially and within the same time interval, the

averaged mobile measurements signify a 15-minute spatiotemporal average along a drive path for a given 15-minute period. In contrast, the averaged model predictions correspond to a spatial average of 15-minute time averages along the same drive path. This inherent difference in our comparison methodology is expected to contribute to limiting the model's ability to capture sharp gradients observed in the measurements.

S1.8 Optimal Sensor Placement Framework

The number of fixed-site sensors in a low-cost sensor network deployment or integrated within a data-driven model, as in the current study, often dictates the cost, scalability, and feasibility of the deployment or model based on the associated data. Recently, there has been a growing focus in the literature on the challenge of sparse sensor placement from the perspective of optimal experiment design, spanning various applications, including air quality sensor placement.

In order to evaluate the impact of the number of *optimally* selected fixed sites on model performance, this study employs the Sparse Sensor Placement for Optimal Reconstruction (SSPOR) algorithm, as outlined by Manohar et al.[7,10]

This method relies on the availability of an oversampled dataset, in this case, an existing dense sensor network, to serve as training data. The algorithm utilizes a combination of QR matrix factorization and singular value decomposition to select a specific number of sites that retain the maximum amount of spatiotemporal variability from all potential sites. In simpler terms, it selects 'n' sites that can best capture the full-scale variability observed in the training data. A detailed explanation of the SSPOR algorithm can be found elsewhere.[7]

Here the model is iteratively constructed using varying numbers of optimally selected sensors, ranging from 10 to 80. Figure S13 demonstrates how changes in the number of fixed-site sensors utilized for model construction affect model accuracy.

S2. SI References

(1) Caubel, J. J.; Cados, T. E.; Preble, C. V.; Kirchstetter, T. W. A Distributed Network of 100 Black Carbon Sensors for 100 Days of Air Quality Monitoring in West Oakland, California. *Environ Sci Technol* **2019**, *53* (13), 7564–7573. https://doi.org/10.1021/acs.est.9b00282.

(2) Chambliss, S. E.; Preble, C. V.; Caubel, J. J.; Cados, T.; Messier, K. P.; Alvarez, R. A.; Lafranchi, B.; Lunden, M.; Marshall, J. D.; Szpiro, A. A.; Kirchstetter, T. W.; Apte, J. S. Comparison of Mobile and Fixed-Site Black Carbon Measurements for High-Resolution Urban Pollution Mapping. *Environ Sci Technol* **2020**, *54* (13), 7848–7857. https://doi.org/10.1021/acs.est.0c01409.

(3) Satopaa, V.; Albrecht, J.; Irwin, D.; Raghavan, B. Finding a "Kneedle" in a Haystack: Detecting Knee Points in System Behavior. In *2011 31st International Conference on Distributed Computing Systems Workshops*; IEEE, 2011; pp 166–171. https://doi.org/10.1109/ICDCSW.2011.20.

(4) Patrick Arnott, W.; Moosmüller, H.; Fred Rogers, C.; Jin, T.; Bruch, R. Photoacoustic Spectrometer for Measuring Light Absorption by Aerosol: Instrument Description. *Atmos Environ* **1999**, *33* (17), 2845–2852. https://doi.org/10.1016/S1352-2310(98)00361-6.

(5) Caubel, J. J.; Cados, T. E.; Kirchstetter, T. W. A New Black Carbon Sensor for Dense Air Quality Monitoring Networks. *Sensors (Switzerland)* **2018**, *18* (3). https://doi.org/10.3390/s18030738.

(6) Apte, J. S.; Messier, K. P.; Gani, S.; Brauer, M.; Kirchstetter, T. W.; Lunden, M. M.; Marshall, J. D.; Portier, C. J.; Vermeulen, R. C. H.; Hamburg, S. P. High-Resolution Air Pollution Mapping with Google Street View Cars: Exploiting Big Data. *Environ Sci Technol* **2017**, *51* (12), 6999–7008. https://doi.org/10.1021/acs.est.7b00891.

(7) Manohar, K.; Brunton, B. W.; Kutz, J. N.; Brunton, S. L. Data-Driven Sparse Sensor Placement for Reconstruction. **2017**. https://doi.org/10.1109/MCS.2018.2810460.

(8) Baraniuk, R. Compressive Sensing [Lecture Notes]. *IEEE Signal Process Mag* **2007**, *24* (4), 118–121. https://doi.org/10.1109/MSP.2007.4286571.

(9) Donoho, D. L. Compressed Sensing. *IEEE Trans Inf Theory* **2006**, *52* (4), 1289–1306. https://doi.org/10.1109/TIT.2006.871582.

(10) Kelp, M. M.; Lin, S.; Kutz, J. N.; Mickley, L. J. A New Approach for Determining Optimal Placement of PM $_{2.5}$ Air Quality Sensors: Case Study for the Contiguous United States. *Environmental Research Letters* **2022**, *17* (3), 034034. https://doi.org/10.1088/1748-9326/ac548f.

(11) Zhou, C.; Gao, M.; Li, J.; Bai, K.; Tang, X.; Lu, X.; Liu, C.; Wang, Z.; Guo, Y. Optimal Planning of Air Quality-Monitoring Sites for Better Depiction of PM $_{2.5}$ Pollution across China. *ACS Environmental Au* **2022**, *2* (4), 314–323. https://doi.org/10.1021/acsenvironau.1c00051.

(12) Brown, S. G.; Eberly, S.; Paatero, P.; Norris, G. A. Methods for Estimating Uncertainty in PMF Solutions: Examples with Ambient Air and Water Quality Data and Guidance on Reporting PMF Results. *Science of The Total Environment* **2015**, *518–519*, 626–635. https://doi.org/10.1016/j.scitotenv.2015.01.022.

(13) Zitnik, M.; Zupan, B. NIMFA: A Python Library for Nonnegative Matrix Factorization. **2018**.

(14) Hoyer, S.; Hamman, J. Xarray: N-D Labeled Arrays and Datasets in Python. *J Open Res Softw* **2017**, *5* (1), 10. https://doi.org/10.5334/jors.148.

(15) The pandas development team. Pandas-Dev/Pandas: Pandas. Zenodo February 2020.
(16) Jordahl, K.; Van den Bossche, J.; Fleischmann, M.; Wasserman, J.; McBride, J.; Gerard, J.; Tratner, J.; Perry, M.; Garcia Badaracco, A.; Farmer, C.; Arne Hjelle, G.; D. Snow, A.; Cochran, M.; Gillies, S.; Culbertson, L.; Bartos, M.; Eubank, N.; Albert, M.; Bilogur, A.; Rey, S.; Ren, C.; Arribas-Bel, D.; Wasser, L.; John Wolf, L.; Journois, M.; Wilson, J.; Greenhall, A.; Holdgraf, C.; Filipe; Leblanc, F. Geopandas/Geopandas: V0.8.1. Zenodo July 2020. https://doi.org/10.5281/zenodo.3946761.
(17) Virtanen, P.; Gommers, R.; Oliphant, T. E.; Haberland, M.; Reddy, T.; Cournapeau, D.; Burovski, E.; Peterson, P.; Weckesser, W.; Bright, J.; van der Walt, S. J.; Brett, M.; Wilson, J.; Millman, K. J.; Mayorov, N.; Nelson, A. R. J.; Jones, E.; Kern, R.; Larson, E.; Carey, C. J.; Polat, İ.; Feng, Y.; Moore, E. W.; VanderPlas, J.; Laxalde, D.; Perktold, J.; Cimrman, R.; Henriksen, I.; Quintero, E. A.; Harris, C. R.; Archibald, A. M.; Ribeiro, A. H.; Pedregosa, F.; van Mulbregt, P.; Vijaykumar, A.; Bardelli, A. Pietro; Rothberg, A.; Hilboll, A.; Kloeckner, A.; Scopatz, A.; Lee, A.; Rokem, A.; Woods, C. N.; Fulton, C.; Masson, C.; Häggström, C.; Fitzgerald, C.; Nicholson, D. A.; Hagen, D. R.; Pasechnik, D. V.; Olivetti, E.; Martin, E.; Wieser, E.; Silva, F.; Lenders, F.; Wilhelm, F.; Young, G.; Price, G. A.; Ingold, G.-L.; Allen, G. E.; Lee, G. R.; Audren, H.; Probst, I.; Dietrich, J. P.; Silterra, J.; Webber, J. T.; Slavič, J.; Nothman, J.; Buchner, J.; Kulick, J.; Schönberger, J. L.; de Miranda Cardoso, J. V.; Reimer, J.; Harrington, J.; Rodríguez, J. L. C.; Nunez-Iglesias, J.; Kuczynski, J.; Tritz, K.; Thoma, M.; Newville, M.; Kümmerer, M.; Bolingbroke, M.; Tartre, M.; Pak, M.; Smith, N. J.; Nowaczyk, N.; Shebanov, N.; Pavlyk, O.; Brodtkorb, P. A.; Lee, P.; McGibbon, R. T.; Feldbauer, R.; Lewis, S.; Tygier, S.; Sievert, S.; Vigna, S.; Peterson, S.; More, S.; Pudlik, T.; Oshima, T.; Pingel, T. J.; Robitaille, T. P.; Spura, T.; Jones, T. R.; Cera, T.; Leslie, T.; Zito, T.; Krauss, T.; Upadhyay, U.; Halchenko, Y. O.; Vázquez-Baeza, Y. SciPy 1.0: Fundamental Algorithms for Scientific Computing in Python. *Nat Methods* **2020**, *17* (3), 261–272. https://doi.org/10.1038/s41592-019-0686-2.
(18) Crameri, F.; Shephard, G. E.; Heron, P. J. The Misuse of Colour in Science Communication. *Nat Commun* **2020**, *11* (1), 5444. https://doi.org/10.1038/s41467-020-19160-7.

S3. SI Tables

Table S1: Sensitivity cases for spatiotemporal model development and evaluation

Model	Data used for model development		Model Performance Evaluation
	LCS data	*Mobile monitoring data*	
**Core**	Utilized data from all 97 near-road fixed sites over the 100-day study period.	Employed time-averaged maps based on 49 days of combined mobile measurements from two cars during the study period.	Model performance assessed against time-varying mobile measurements from both vehicles.
**Sensitivity Case A**	Employed data from all 97 near-road fixed sites over the 100-day period.	Utilized time-averaged maps from mobile measurements made by either car 1 or car 2 during the study period.	Model performance evaluated against time-varying mobile measurements from the car not included in model development.
**Sensitivity Case B**	Randomly selected 70% of LCS sites repeatedly over 1000 iterations.	Utilized time-averaged maps based on 49 days of combined mobile measurements from two cars.	Model performance evaluated against 30% of LCS sites held-out from model development across 1000 iterations.
**Sensitivity Case C**	Varied the number of LCS sites (10 to 80 sites, in increments of 5; 1000 iterations each).	Utilized time-averaged maps based on 49 days of combined mobile measurements from two cars.	Model predicted LCS time series for the remaining LCS sites across 16000 iterations.
**Sensitivity Case D**	Utilized data from all 97 near-road fixed sites over the 100-day period.	Employed time-averaged maps based on 49 days of mobile measurements conducted outside the study period (100 random trials).	Model performance assessed against time-varying mobile measurements from both cars during the study period.
**Sensitivity Case E**	Utilized data from all 97 near-road fixed sites over the 100-day period.	Varied the number of input pollutant maps between n= 4 to 10 (including BC) used to build the model. Tested each possible combination of subsampling n pollutants from the total set of 10 pollutants.	Model performance assessed against time-varying mobile measurements from both vehicles.

S4. SI Figures

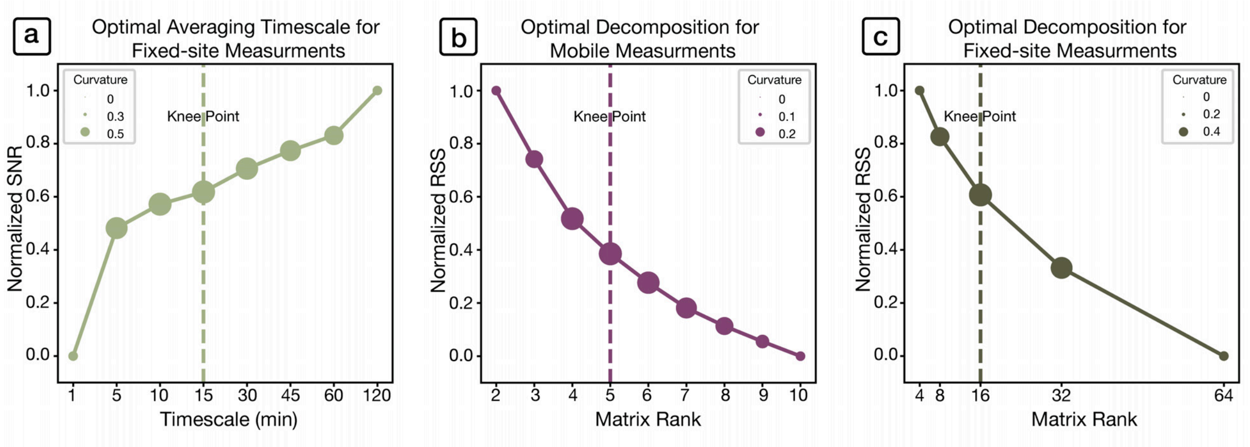

Figure S1: Optimizing Averaging Time Scale and Factorization Rank Selection.
(a) The relationship between Signal-to-Noise Ratio (SNR) and increasing averaging time scale for fixed-site measurements is examined to determine the optimal temporal aggregation scale. We find the knee point on this curve an averaging time of 15 minutes, which aligns with the findings of Chambliss et al.[2] Consequently, we selected 15 minutes as the optimal averaging time scale for the fixed-site data. **(b)** The variability of residual sum of squares for the *pollutant-location* system ($\mathbf{X}_{\boldsymbol{M}}$) is investigated as the number of factors in the matrix factorization process is increased. The knee point on this curve is observed at 5, leading to the selection of 5 as the optimal rank for decomposing the $\mathbf{X}_{\boldsymbol{M}}$ matrix. **(c)** Similarly, for the *location-time* system ($\mathbf{X}_{\boldsymbol{F}}$), representing fixed-site BC measurements, the residual sum of squares is analyzed across different factorization ranks. The knee point on this curve is identified at 16, indicating that 16 is the optimal rank for factorizing the $\mathbf{X}_{\boldsymbol{F}}$ matrix. These optimized parameters are essential for the effectiveness of the proposed model, as discussed in detail in Section 2.2. In each case (a-c), we used the Python package `kneed` to identify the knee-point.

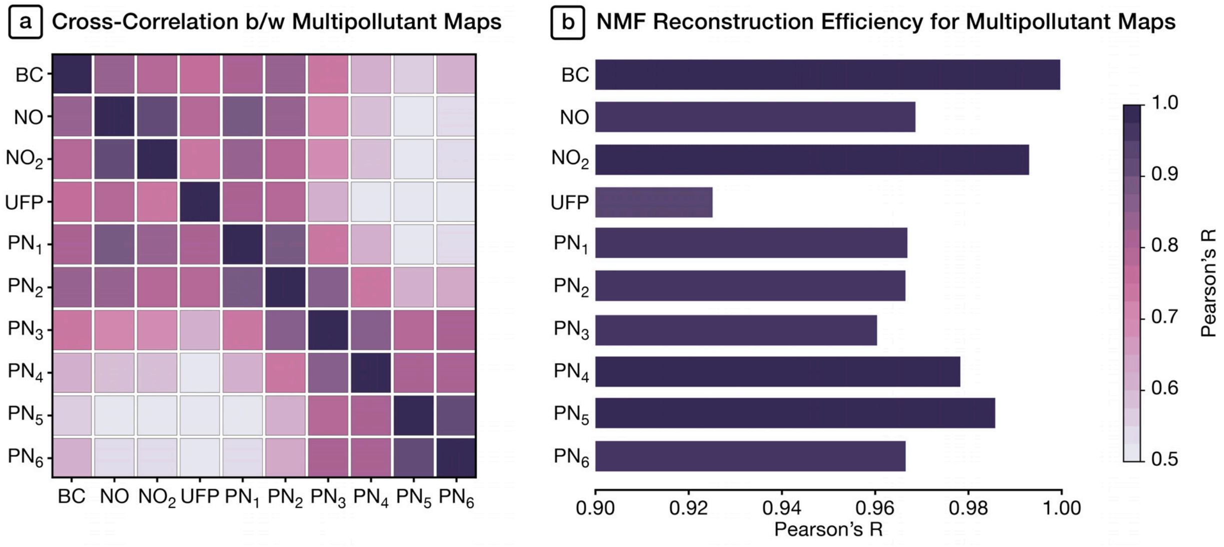

Figure S2: Cross-correlation and NMF Reconstruction Efficiency for Time-Averaged Multipollutant Maps

(a) Spatial cross-correlation among various time-averaged pollutant maps (BC, NO, NO_2, UFP, PN_1, PN_2, PN_3, PN_4, PN_5, PN_6) derived from routine mobile measurements. This panel illustrates the degree of similarity or dissimilarity between different pollutants across the spatial domain. A higher correlation indicates a stronger spatial relationship between two pollutants, suggesting potential shared emission sources or similar spatial distribution patterns. (b) Pearson's correlation coefficient (R) between the Non-negative Matrix Factorization (NMF) based reconstruction and the original input multipollutant spatial maps. The higher the correlation coefficient, the better the NMF model reconstructs the spatial variability observed in the input pollutant maps. This comparison serves as a proxy for the degree of shared covariance or communality among pollutants, indicating the extent to which they can be expressed as a combination of others in the set. Note that in particular, we find $R = 0.995$ for our pollutant of interest, BC.

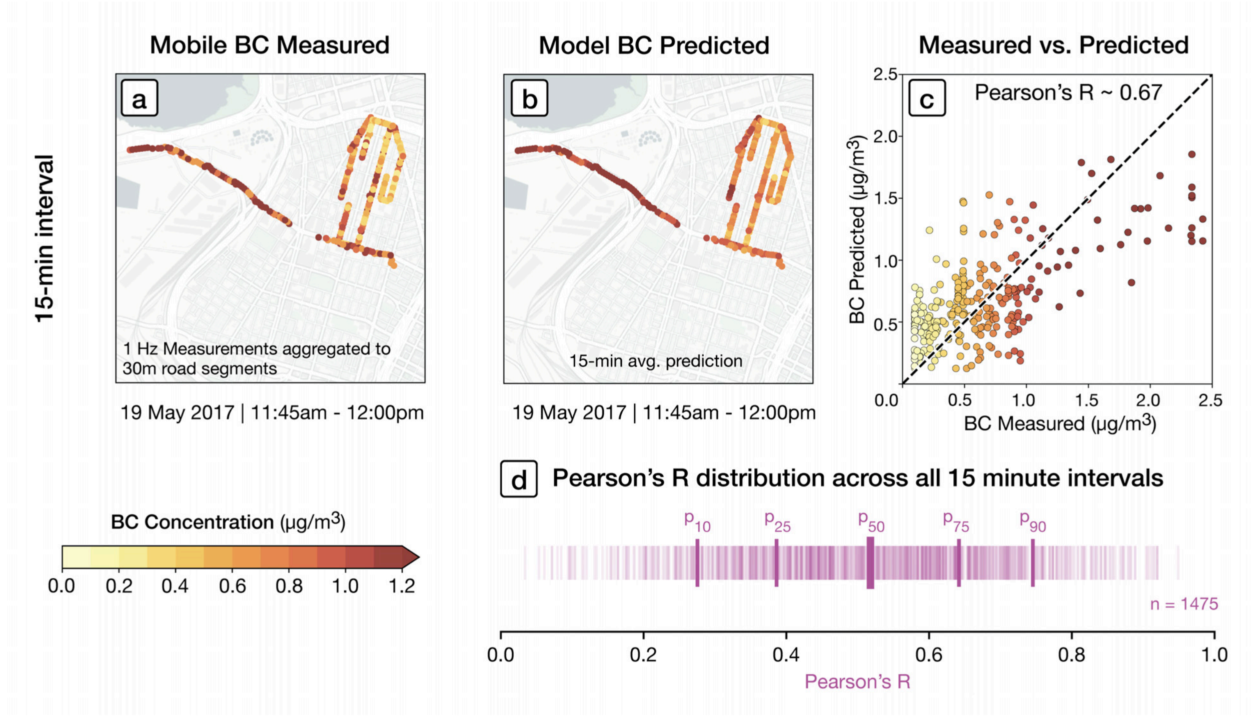

Figure S3: Spatiotemporal alignment between mobile measurements and model output

(a) Mobile measurements (1 Hz) obtained during a single example 15-minute interval, aggregated to 30m road segments, and **(b)** 15-minute average model predictions for road segments traversed by the car during the same time interval. Despite differences in temporal resolution, **(c)** reveals a Pearson's correlation coefficient of R = 0.67 between the two datasets for this example. Similar comparisons extend to 1475 distinct 15-minute periods, with the distribution of R depicted in **(d)**, ranging between 0.28 and 0.75 (10th and 90th percentiles, respectively), and a median value of 0.52.

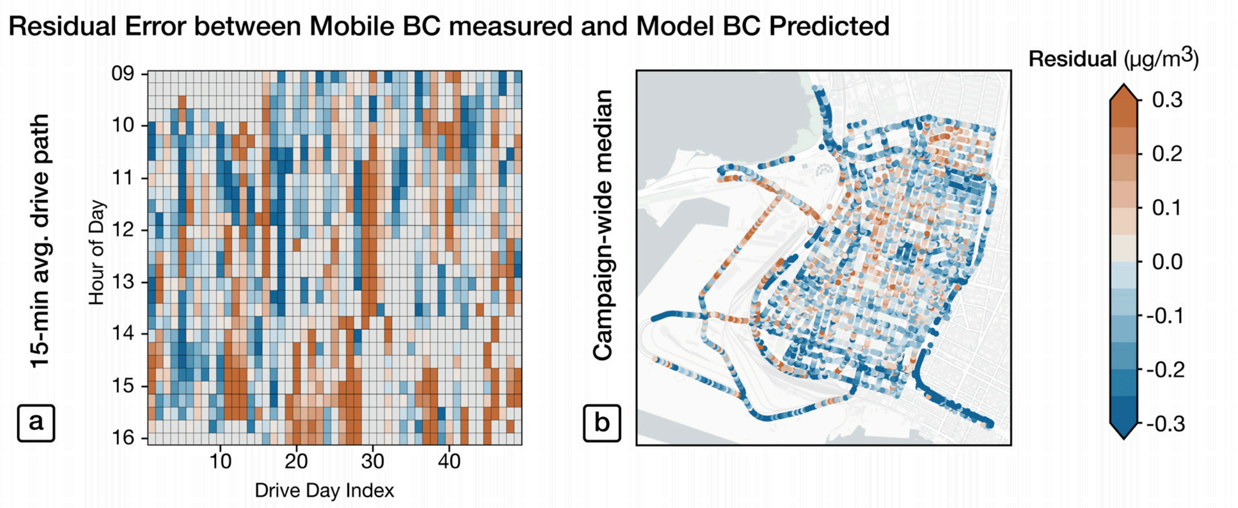

Figure S4: Residual Analysis of Mobile BC Measurements vs. Model Predictions
In Figure 2b-c, one-to-one comparisons demonstrate strong correlations between mobile BC measurements and the model's BC concentration predictions Figure 2b-c (*right*). Here, we assess the spatial patterns of these residuals. **(a)** Discrepancies between mobile BC measurements and model predictions when averaged along 15-minute drive paths (difference between Figure 2b (*left & center*)). **(b)** Deviations between mobile BC measurements and model predictions when spatially aggregated (difference between Figure 2c (*left & center*)). Both panels offer insights into the nature of the differences between observed and predicted BC concentrations. These analyses indicate that the peak absolute error typically falls within the order of approximately 25%. Moreover, the evaluation demonstrates that the errors are randomly distributed, with no discernible temporal **(a)** or spatial bias **(b)**.

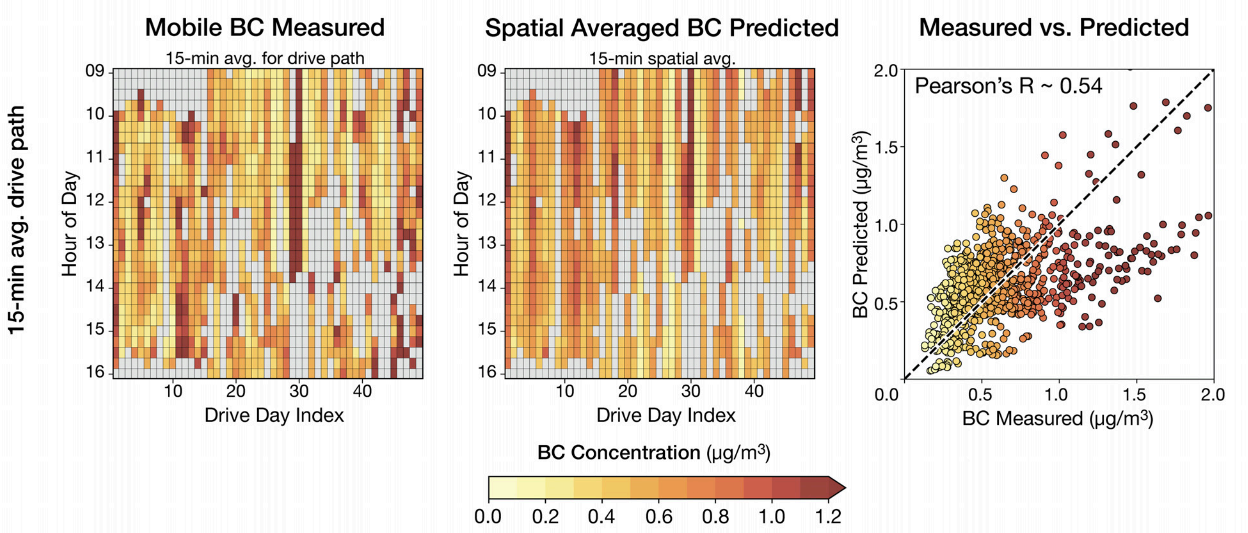

Figure S5: Assessing fine-scale versus regional variability captured by the spatiotemporal model. In Figure 2a, we evaluate the spatiotemporal model's proficiency in capturing variations along a Lagrangian drive path of mobile measurements. Here, we aim discern the extent to which our model captures true *spatiotemporal* variability, rather than simply capturing temporal variability. This figure presents a comparison of the 15-minute spatiotemporal averages along the sampled drive path for mobile measurements (*left*) and the corresponding spatially averaged model predictions (*center*) on days featuring mobile sampling during the campaign. Notably, the spatially averaged model output exhibits a relatively weaker correlation with observations ($R = 0.54$, *right*). This is in contrast to the spatiotemporal model output, as showcased in Figure 2a, which captures both the spatial and temporal components at higher fidelity ($R = 0.77$). The overall interpretation of this assessment is that our core model captures fine-grained, spatially localized concentration patterns at *individual* time steps that are not captured by the temporal patterns of the spatial average across the domain.

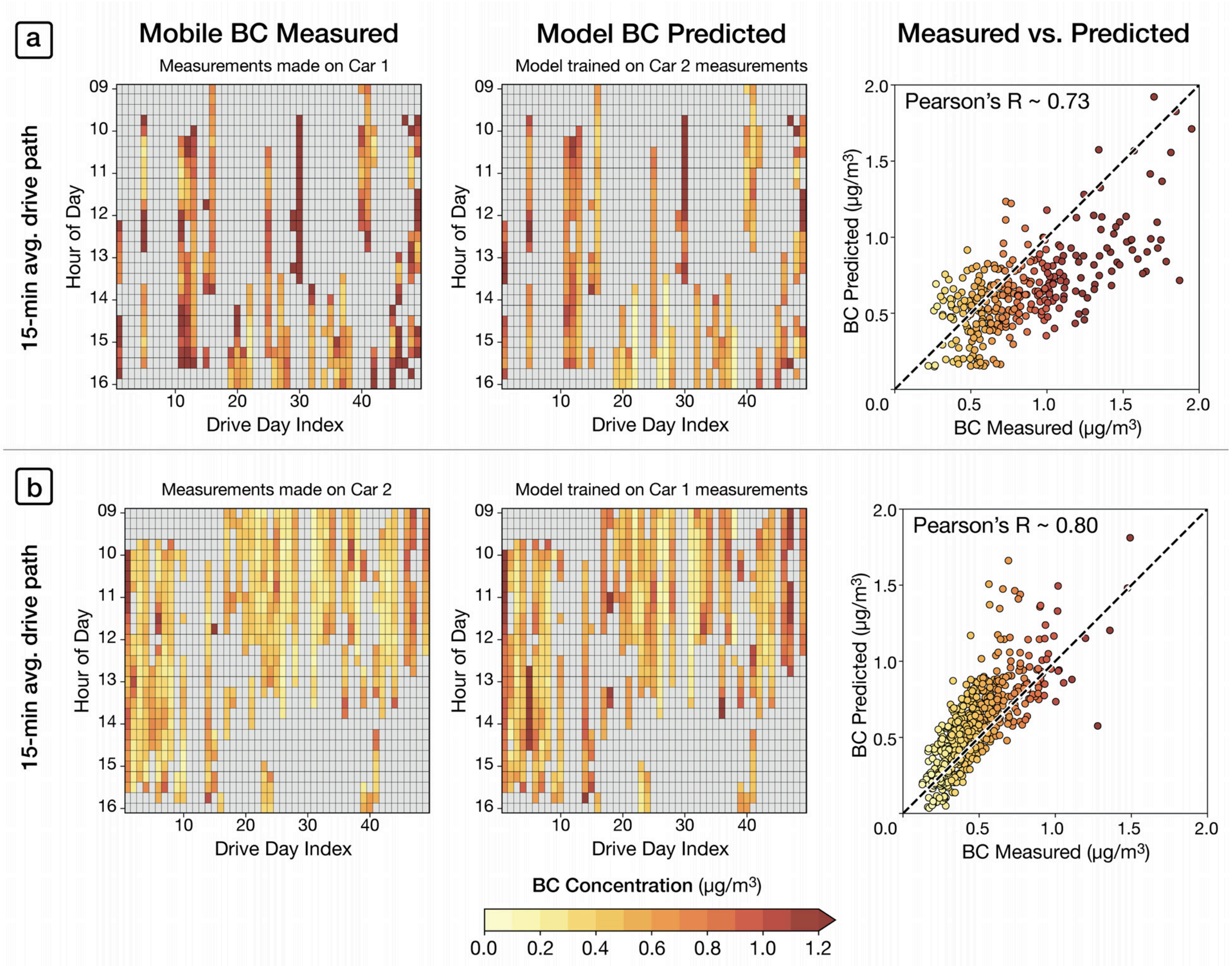

Figure S6: Robustness to temporal sampling bias due to mobile lab driving patterns (Sensitivity Case A). The model's reliance on time-averaged mobile measurement maps assumes representativeness of true temporal averages. However, variations in mobile lab driving patterns introduce potential biases. The core model incorporates data from two distinct mobile labs. To assess the possible influence of a single vehicle's driving pattern, we limit the model to using measurements from a single mobile lab and evaluate its capacity to predict measurements from the other mobile lab. **(a)** Comparison of 15-minute spatiotemporal averages along the sampled drive path for mobile measurements from car 1 (*left*), model predictions (*center*) using car 2 measurements, and the correlation between the two (*right*, Pearson's R = 0.73). This analysis indicates a slight underprediction of peak values by the model in comparison to true measurements. **(b)** Comparison of 15-minute spatiotemporal averages along the sampled drive path for mobile measurements (*left*), model predictions (*center*), and the correlation between the two (*right*, Pearson's R = 0.8). In this case, the model is constructed using car 1 data and tested against car 2 measurements, revealing a slight overprediction of peak values. These discrepancies in (a) and (b) are influenced by instrument biases, drive patterns, and specific data collection days. However, both scenarios (a) and (b) highlight the model's consistent ability to capture general measurement patterns, demonstrating its resilience to temporal sampling bias associated with varying mobile lab driving patterns.

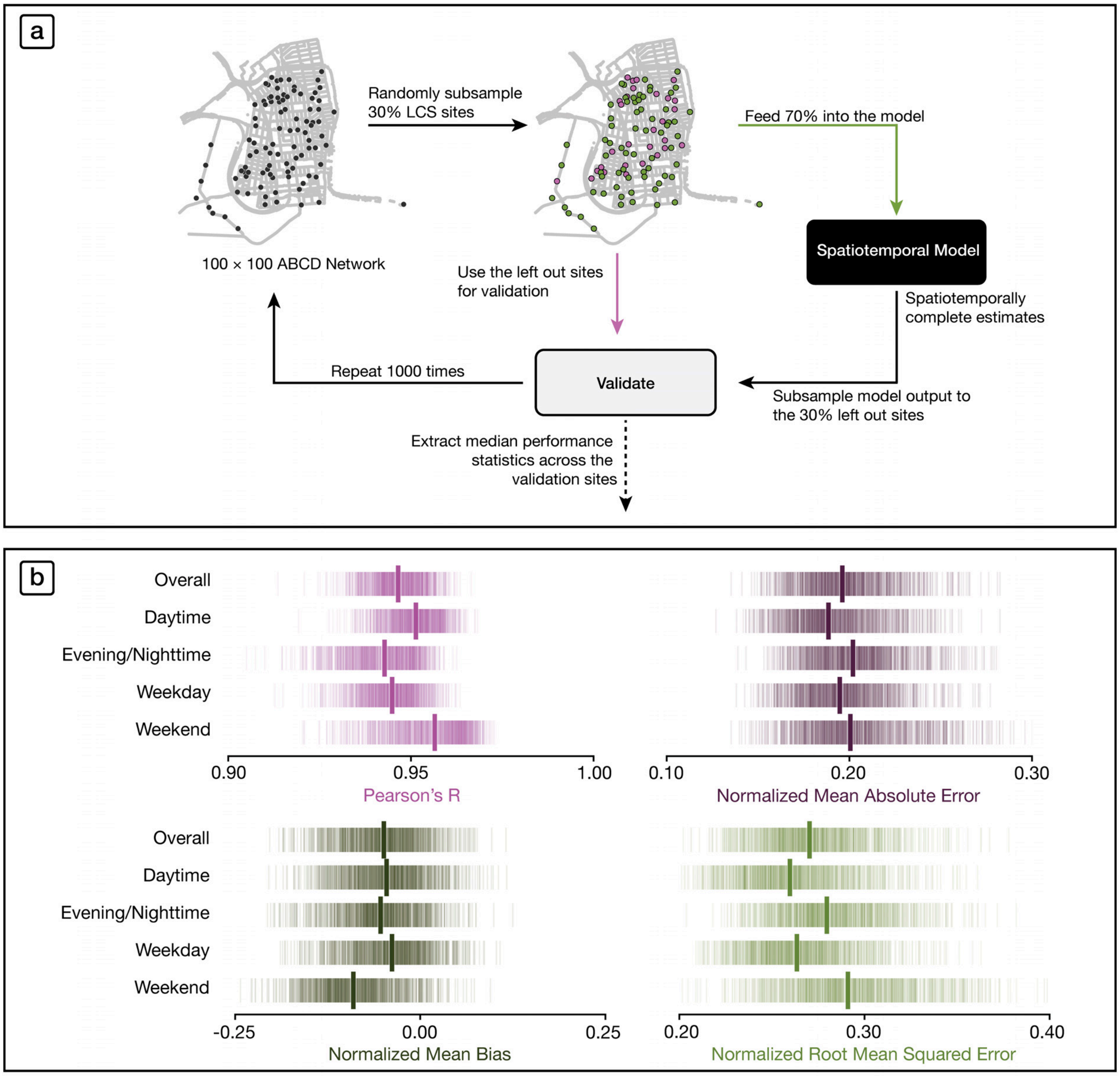

Figure S7: Validation of model time-series performance using out-of-sample fixed-site measurements (Sensitivity Case B). (a) We validated out-of-sample prediction performanceat fixed sites using , a random subsampling approach is employed. In 1000 random trials, we selected approximately 70% of the 97 fixed-site sensor (68 sites) for model-building, with the remaining 29 reserved for model evaluation. Time-resolved model predictions for these 29 out-of-sample sites are compared against LCS measurements at their respective locations, assessing the model's temporal performance using the Pearson's R correlation coefficient and error statistics NMAE, NMB, and NRMSE. **(b)** Time-series performance statistics for each of the 1000 validation trials, quantifying the performance the model at predicting BC time series. We quantify this time-series metric for each of the 29 out-of-sample sites and report here the spatial median over 29 sites. Bold vertical lines indicate the median over 1000 draws. The results demonstrate the robustness and reliability of the model's performance even during periods with limited mobile monitoring, such as on nights and weekends.

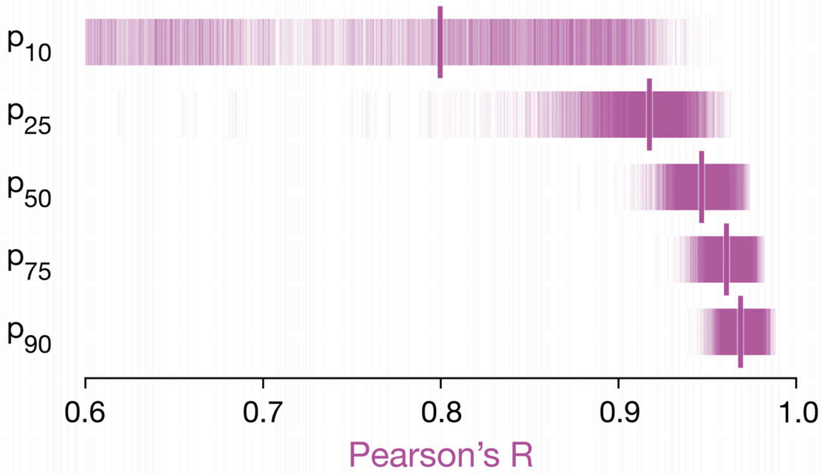

Figure S8: Spatial variability in model temporal Pearson's *R* for out-of-sample estimation of fixed-site measurements. As a counterpart to Figure S6b, which illustrates the spatial median of the time-series performance of our model at 29 randomly chosen out-of-sample evaluation sites, we report here other moments the spatial distribution of temporal performance for the core "overall" evaluation. This figure provides the spatial 10th, 25th, 75th, and 90th percentiles for the model's temporal *R* in each of the 1000 trials. Additionally, we reproduce the overall result from Figure 6b (p_{50}), which reflects the median performance among all 29 sites. In each case, the median value across the 1000 trials is bolded. The 10th and 90th percentiles in this plot correspond to approximately the 3rd worst and the 3rd best out of the 29 temporal Pearson's *R* for each of the 1000 trials. A key insight from this analysis is that out-of-sample temporal prediction performance for the model is moderately diminished for a small number of LCS sites in the domain, with a median temporal *R* among 1000 draws of 0.80 at the 10th percentile site instead of 0.94 for the median site. The temporal predictive performance at the poorest-performing sites is also noticeably less stable among random draws of training/test sites. This result suggests that there are a small number of locations in the domain with diurnal profiles that can be difficult to predict if the model happens to be trained with LCS data from other very dissimilar locations.

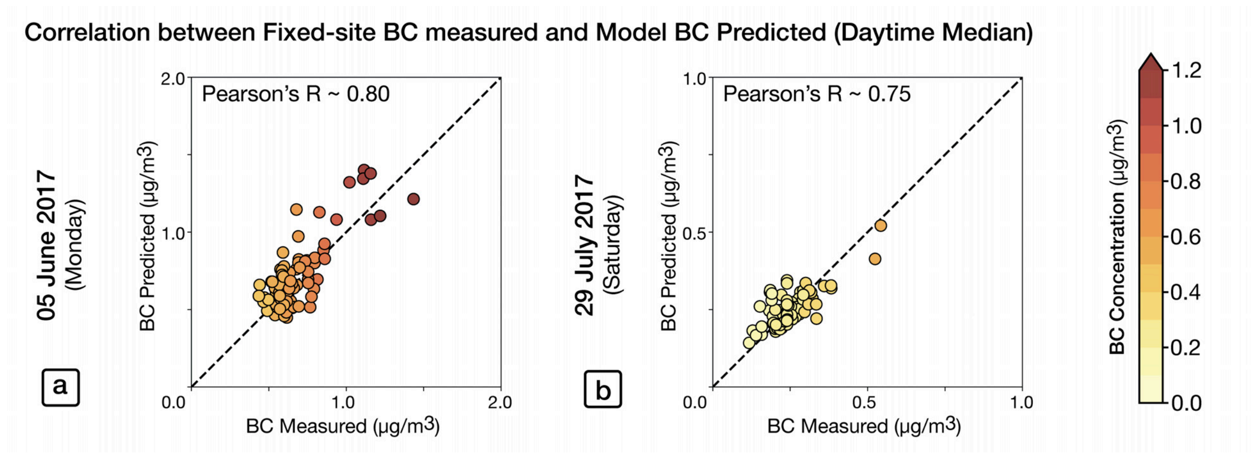

Figure S9: Correlation between Modeled and Observed Fixed-Site BC Concentrations
As shown in Figure 3b-c, the model's spatiotemporal completeness enables the filling of data gaps between LCS sites, revealing extensive spatial variability in black carbon (BC) concentrations on any given day. Here, we emphasize that, even on specific days, the daily median model BC predictions at LCS locations closely matches the median BC measurements at the LCS sites. **(a)** Demonstrates the strong correlation (Pearson's R = 0.8) between daytime median fixed-site BC measurements and model predictions for June 5th, 2017 (correlation between Figure 3 (b, center & right)). **(b)** Highlights a substantial correlation (Pearson's R = 0.75) between daytime median fixed-site BC measurements and model predictions for July 29th, 2017 (correlation between Figure 3b (center & right)).

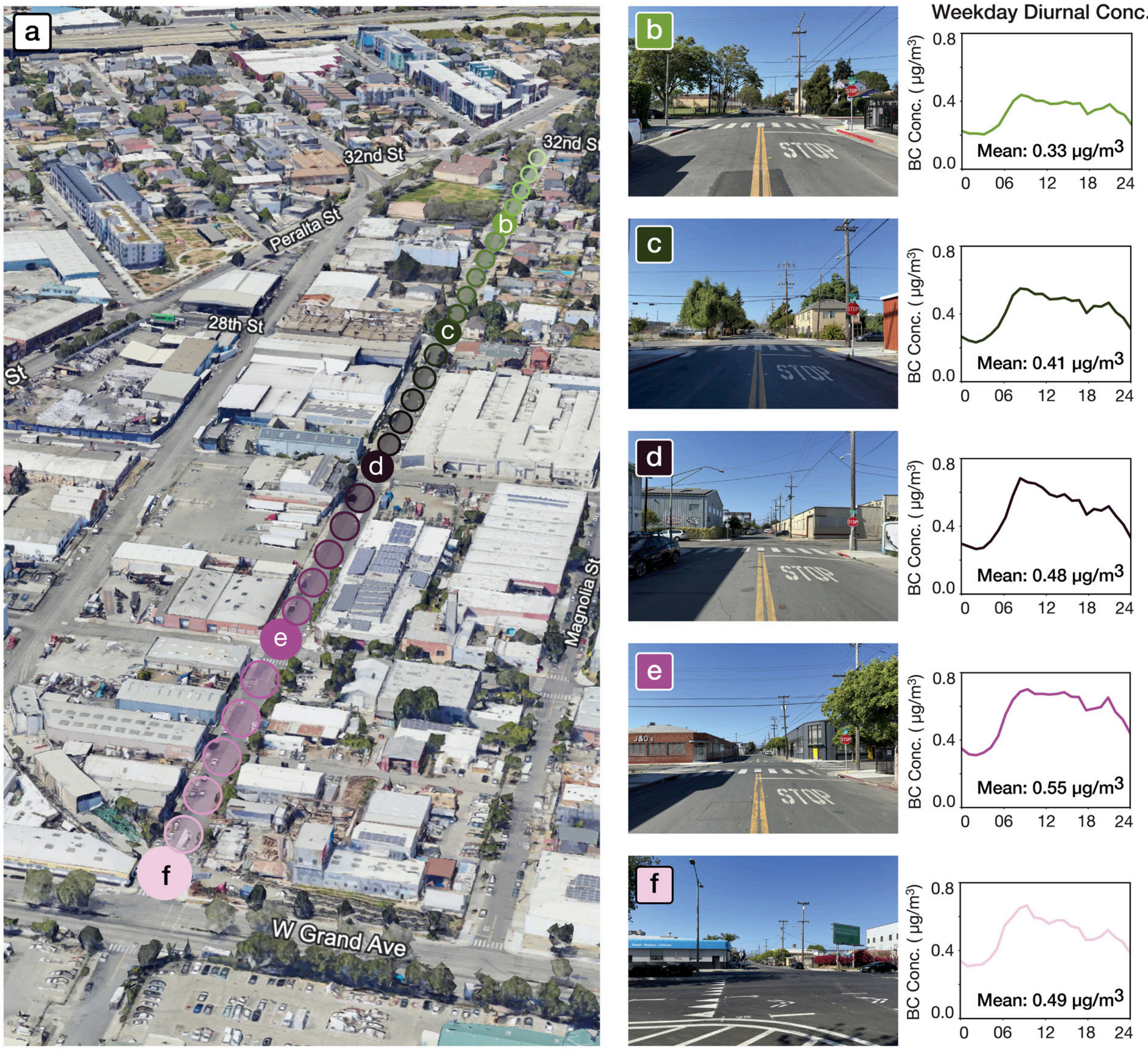

Figure S10: Aerial and street view perspectives of the Union Street transect (*cf.* Figure 4f-g). (a) Aerial view of the Union Street transect highlighted in Figure 4f-g, featuring the zoomed-in neighborhood detailed in Figure 4c-e). **(b-f)** Street View images taken within ~ 6 months of the measurement campaign at each intersection along Union Street, accompanied by the median diurnal profile and average weekday concentrations at each location. (Map data © 2023 Google).

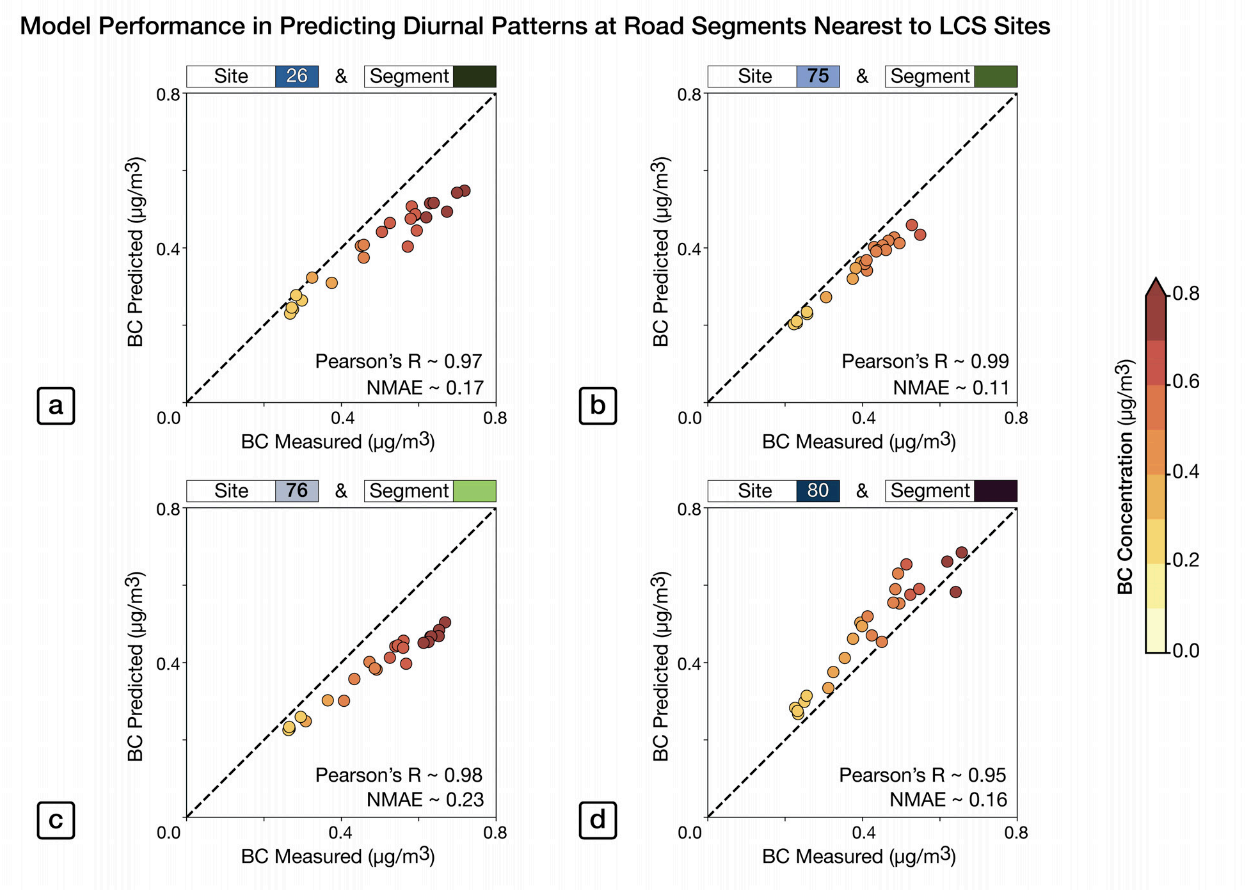

Figure S11: Model Performance in Predicting Diurnal Patterns at Road Segments Nearest to LCS sites.

As illustrated in Figure 4f-g, the model output shows spatial variability in the diurnal patterns of black carbon (BC) concentrations along a 1km spatial transect. That figure illustrates how the model fills key spatial gaps along this transect that were not covered by the four fixed-site sensors in this area. Here, we additionally demonstrate that the model output for the road segments closest to these four LCS sites closely matches the measured diurnal profiles at these four sites. Subfigures **(a-d)** demonstrate the robust correlation and low Normalized Mean Absolute Error (NMAE) between **(a)** Site 26 (Pearson's R = 0.97, NMAE = 0.17), **(b)** Site 75 (Pearson's R = 0.99, NMAE = 0.11), **(c)** Site 76 (Pearson's R = 0.98, NMAE = 0.23), and **(d)** Site 80 (Pearson's R = 0.95, NMAE = 0.16) with the model-predicted BC concentrations at the road segments nearest to the respective monitoring sites.

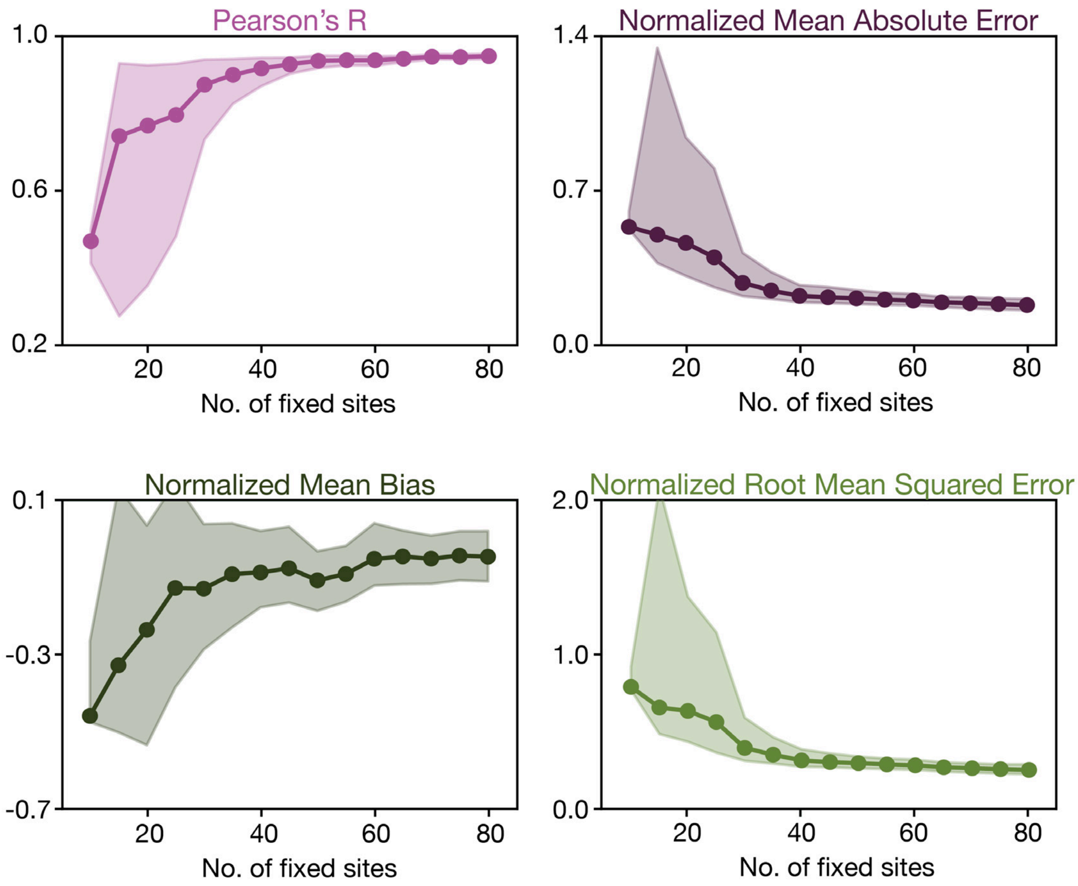

Figure S12: Impact of fixed-site sampling intensity (randomly chosen) on model performance (Sensitivity Case C). The core model is constructed using data from all 97 fixed-site sensors. This evaluation investigates the influence of sensor density, termed sampling intensity, on the performance of the spatiotemporal model. The model is iteratively constructed with varying sampling intensities, with 1000 random draws for each scenario. The figure depicts how changes in the number of fixed-site sensors used for model construction impacts model accuracy. Performance variation is presented in terms of Pearson's *R*, NMAE, NMB, and NRMSE, as a function of sampling intensity. Similar to Sensitivity Case B in Figure S7, each metric represents the median temporal performance across sites not included in model development. The bolded line signifies the median of this temporal performance across the 1000 iterations, while the confidence intervals depict the 10th and 90th percentiles across these iterations. The observed trends across all four metrics suggest that model performance stabilizes as the number of sensors approaches the range of 30-40 sensors. Beyond this threshold, all evaluation metrics reach a nearly constant value. This observation underscores the significance of selecting an optimal network configuration with approximately 30 sensors. It strikes a balance between network density, modeling accuracy, and resource efficiency, highlighting the practical considerations in fixed-site sensor network design.

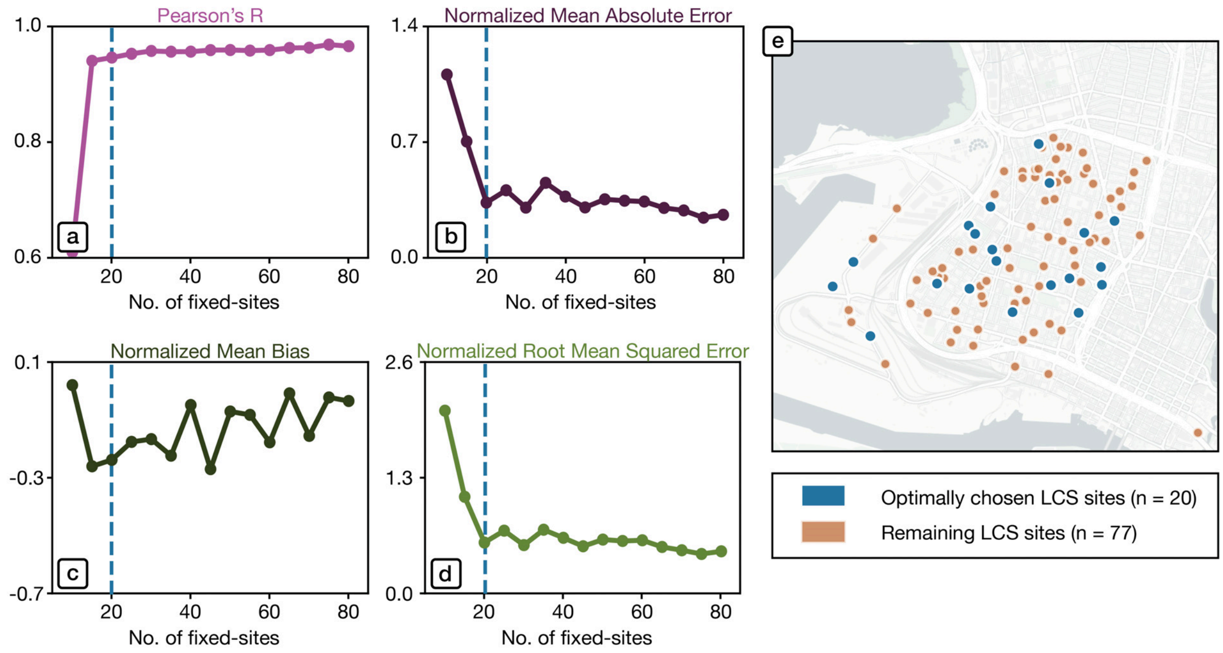

Figure S13: Impact of fixed-site sampling intensity on model performance. The core model utilizes data from all 97 fixed-site sensors, while Sensitivity Case C assesses the impact of sampling intensity on model performance by randomly subsampling sensors. This evaluation investigates the influence of sampling intensity on model performance when sensors are optimally chosen using the Sparse Sensor Placement for Optimal Reconstruction (SSPOR) algorithm, based on singular value decomposition (SVD) and QR pivoting, as presented by Manohar et al.[7] The model is iteratively constructed with varying number of sensors ranging from 10 to 80. The figure illustrates how changes in the number of fixed-site sensors used for model construction, impact model accuracy. Performance variation is presented in terms of **(a)** Pearson's R, **(b)** NMAE, **(c)** NMB, and **(d)** NRMSE, as a function of sampling intensity. Similar to Sensitivity Case B in Figure S6, each metric represents the median temporal performance across sites not included in model development. The observed trends across all four metrics suggest that while approximately 30-40 randomly chosen sensors are sufficient for robust model performance, around 20 optimally chosen sensors are adequate for achieving the same level of accuracy in model performance. Panel **(e)** illustrates the subset of 20 optimally chosen LCS sites from the original 97 LCS sites, as indicated optimal by the SSPOR algorithm. These optimal sites are concentrated toward the port and industrial area, where past studies have observed high BC concentration and variability.[1]

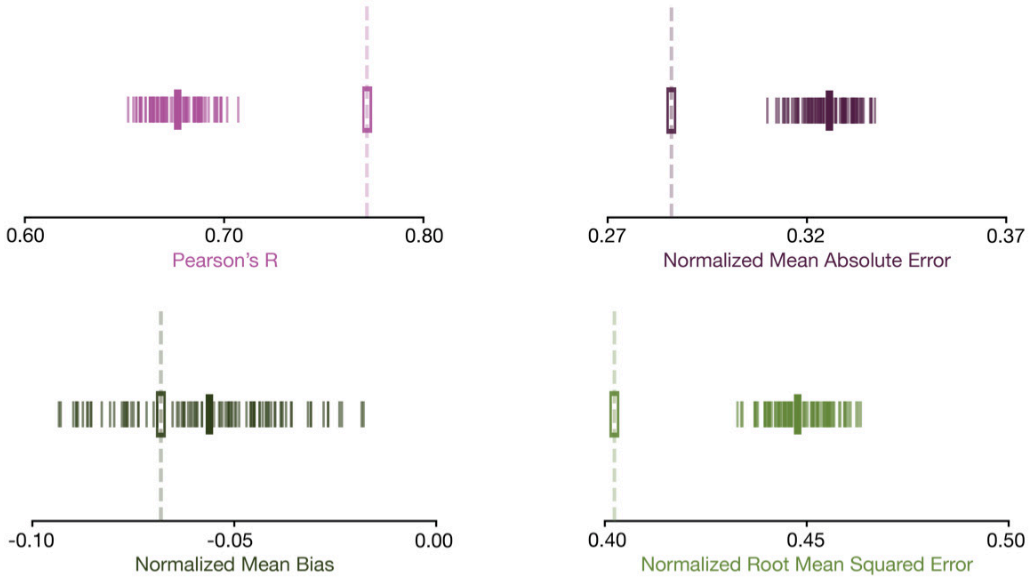

Figure S14: Assessing model performance with mobile measurements from different time periods (Sensitivity Case D). Sensitivity case D evaluates the model's robustness when predicting mobile measurements during a campaign using time-averaged maps derived from a period outside the fixed-site measurements' timeframe. The model is iteratively developed over 100 draws, incorporating time-averaged maps randomly chosen from mobile sampling days outside the fixed-site measurements' timeframe while maintaining the same mobile sampling intensity (i.e., 49 days between May 2015 to Dec 2017 excluding the 100-day study period in 2017) as in the core model. Model performance is assessed by comparing model predictions against the 15-minute spatiotemporally averaged mobile measurements presented in Figure 2a (*left*). The figure presents performance statistics, including Pearson's *R*, NMAE, NMB, and NRMSE, for each of the 100 trials. Additionally, median values for these performance metrics are highlighted, offering a concise overview of model accuracy and consistency under diverse random subsampling scenarios. The dashed lines indicate the core model's performance for the respective metrics. It is noteworthy that, with the exception of NMB, all other metrics indicate poorer performance in the Sensitivity Case D trials compared to the core model. This investigation underscores the model's capacity to discern features and provide accurate estimates as long as the time-averaged patterns from different time periods align with those of the campaign. Notably, significant changes in the time-averaged pattern may limit the model's ability to capture alterations in mobile measurements from another time period.

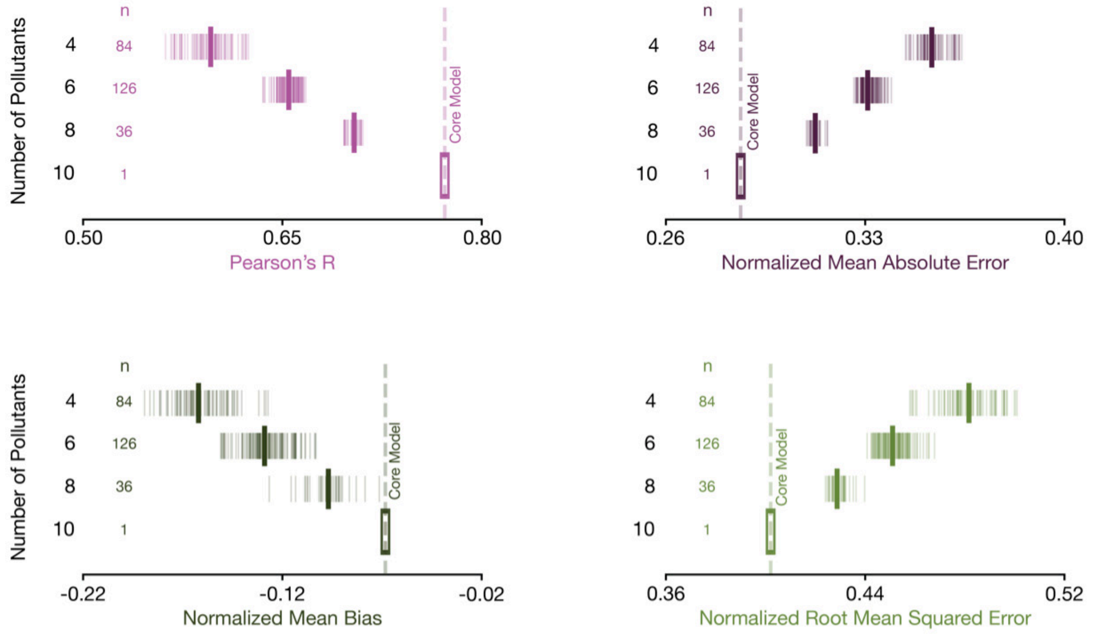

Figure S15: Assessing model performance with varying number of pollutants measured with mobile sampling (Sensitivity Case E). The core model utilizes time-averaged maps derived from mobile measurements for all 10 pollutants measured by the mobile platform. Sensitivity Case E evaluates the model's performance based on the number of pollutants measured, thereby varying the number of time-averaged pollutant maps input to the model in addition to BC. The model is iteratively constructed for the number of pollutants (including BC) ranging from 4 (i.e., BC + 3 randomly selected pollutants) to 10 (all pollutants). For each selected 'n' number of pollutants, all possible combinations of subsampling 'n' from the 10 were investigated. For example, 84 combinations (${}^{1}C_{1} \times {}^{9}C_{3}$) were explored for choosing 4 pollutants (including BC), 126 combinations (${}^{1}C_{1} \times {}^{9}C_{5}$) for choosing 6, and 36 combinations (${}^{1}C_{1} \times {}^{9}C_{7}$) for choosing 8. The figure presents performance statistics, including Pearson's R, NMAE, NMB, and NRMSE, for each of the combinations. Additionally, median values for these performance metrics are highlighted, offering a concise overview of model accuracy and consistency under all subsampling scenarios. The dashed lines indicate the core model's performance for the respective metrics. Note that all metrics indicate a steady improvement in median model performance with an increasing number of pollutants measured. This improvement can be attributed to the increased number of pollutants (and their shared covariance), enabling the non-negative matrix factorization (NMF) to better resolve a higher number of pollutant-invariant patterns, which in turn capture more spatial variability and improve the model's ability to fill in the gaps in the time-invariant patterns.
